# Integrated characterisation of nine species of the Schistorchiinae (Trematoda: Apocreadiidae) from Indo-Pacific fishes: two new species, a new genus, and a resurrected but ‘cryptic’ genus

**DOI:** 10.1007/s11230-023-10093-5

**Published:** 2023-05-10

**Authors:** Lori Magro, Scott C. Cutmore, Maite Carrasson, Thomas H. Cribb

**Affiliations:** 1 School of Biological Sciences, The University of Queensland, St Lucia, QLD 4072 Australia; 2 Queensland Museum, Biodiversity and Geosciences Program, South Brisbane, QLD 4101 Australia; 3 Departament de Biologia Animal, de Biologia Vegetal i d’Ecologia, Universitat Autònoma de Barcelona, Cerdanyola del Vallès, 08193 Barcelona, Spain

## Abstract

We report nine species of the Schistorchiinae Yamaguti, 1942 (Apocreadiidae Skrjabin, 1942) from Indo-Pacific marine fishes. Molecular data (ITS2 and 28S rDNA and *cox*1 mtDNA) are provided for all species and the genus-level classification of the subfamily is revised. For *Schistorchis* Lühe, 1906, we report the type-species *Sch. carneus* Lühe, 1906 and *Sch. skrjabini* Parukhin, 1963. For *Sphinteristomum* Oshmarin, Mamaev & Parukhin, 1961 we report the type-species, *Sph. acollum* Oshmarin, Mamaev & Parukhin, 1961. We report and re-recognise *Lobatotrema* Manter, 1963, for the type and only species, *L. aniferum* Manter, 1963, previously a synonym of *Sph. acollum*. *Lobatotrema aniferum* is phylogenetically distant from, but morphologically similar to, *Sph. acollum* and *Lobatotrema* is recognised as a ‘cryptic genus’. We propose *Blendiella* **n. gen.** for *B. trigintatestis* **n. sp.** and *B. tridecimtestis* **n. sp.** These species are broadly consistent with the present morphological concept of *Schistorchis* but are phylogenetically distant from the type-species; a larger number of testes and some other subtle morphological characters in species of *Blendiella* serve to distinguish the two genera. We report three species of *Paraschistorchis* Blend, Karar & Dronen, 2017: *P. stenosoma* (Hanson, 1953) Blend, Karar & Dronen, 2017 (type-species), *P. seychellesiensis* (Toman, 1989) Blend, Karar & Dronen, 2017, and *P. zancli* (Hanson, 1953) Blend, Karar & Dronen, 2017. *Lobatotrema aniferum, P. stenosoma,* and *Sch. carneus* each have two distinct *cox*1 populations either over geographical range or in sympatry. Available evidence suggests that most of these species, but not all, are widespread in the tropical Indo-Pacific.

## Introduction

Members of the Apocreadiidae Skrjabin, 1942 parasitise a wide range of marine and freshwater fishes, with the family comprising four subfamilies (the Apocreadiinae Skrjabin, 1942; Megaperinae Manter, 1934; Postporinae Yamaguti, 1958; and Schistorchiinae Yamaguti, 1942) and 23 genera (Blend et al., 2017). The family name is presently controversial. Blend et al. (2017) proposed that the senior synonym is Megaperidae Manter, 1934, but commentary in WoRMS (2022) argues for the retention of Apocreadiidae (citing that the change was non-compliant with Article ICZN Article 35.5). For the present, we accept the latter, arguably conservative view. Morphological characters uniting species within this family include an I-shaped excretory vesicle, lack of a cirrus-sac, extensive vitelline follicles, dispersed eye-spot pigment in the forebody, and the genital pore opening immediately anterior or (rarely) posterior to the ventral sucker (Cribb & Bray, 1999). Although this morphology is far from distinctive, molecular phylogenetic analysis has consistently shown the family to be monophyletic and indeed worthy of recognition as the sole family in the suborder Apocreadiata (Olson et al., 2003; Pérez-Ponce de León & Hernandez-Mena, 2019).

The subfamily Schistorchiinae is presently recognised within the Apocreadiidae by the possession of an unusual and distinctive U-shaped partial sphincter embedded within the oral sucker (Cribb, 2005; Pulis et al., 2014). Following recent revision by Blend et al. (2017), the subfamily is recognised as comprising six genera and 17 species, all of which infect fishes of the Indo-west Pacific except for the enigmatic species *Sphincteristomum mediterraneae* Abid-Kachour, Mouffok & Boutiba, 2013, which parasitises sparid fishes in the Mediterranean Sea (Abid-Kachour et al., 2013).

Here we report on schistorchiines from Indo-Pacific fishes incorporating multi-loci molecular data that enables independent consideration of the genus-level classification proposed by Blend et al. (2017).

## Materials and methods

### Collecting

Fishes were collected by spear, seine net, tunnel net or line from localities off Australia, French Polynesia, New Caledonia, and Palau. Digeneans were collected from freshly killed fish as described by Cribb & Bray (2010), fixed by being pipetted into nearly boiling saline, and immediately preserved in either formalin (early work) or 80% ethanol (recent work). Some individual worms preserved in 80% ethanol were processed for parallel morphological and molecular analyses (hologenophores and paragenophores *sensu* Pleijel et al., 2008).

### Morphology

Trematodes for morphological examination were washed in distilled water, stained in Mayer’s haematoxylin, destained in 1.0% hydrochloric acid, and neutralised in 1.0% ammonium hydroxide. Worms were then dehydrated in a graded ethanol series, cleared in methyl salicylate, and mounted on slides in Canada balsam. Measurements were taken from a live feed produced with an Olympus SC50 digital camera attached to an Olympus BX-53 compound microscope with cellSens v1.13 software. Drawings were made using a drawing tube connected to the same microscope and subsequently digitised using a drawing pad and Adobe Illustrator CC 2018. Figures are presented for species or combinations not previously reported from Australia. All measurements are in micrometres and given as the range, followed by the mean in parentheses. The following abbreviations are used: MNHN, Museum National d’Histoires Naturelles, Paris, France; QM, Queensland Museum, Brisbane, Australia; WAM, Western Australian Museum, Perth, Australia. To comply with the recommendations set out in article 8.5 of the amended 2012 version of the International Code of Zoological Nomenclature (ICZN, 2012), details of the new species have been submitted to ZooBank and registered with Life Science Identifiers (LSID), which are provided in the taxonomic summaries.

### Molecular analyses

Specimens for molecular analysis were processed according to the protocols used by Cribb et al. (2022). Three genetic markers were sequenced, the second internal transcribed spacer region (ITS2 rDNA), the large (28S) ribosomal subunit RNA coding region and the cytochrome c oxidase subunit 1 (*cox*1 mtDNA) mitochondrial region. The complete ITS2 region was amplified and sequenced using the primers 3S (Morgan & Blair, 1995) and ITS2.2 (Cribb et al., 1998), the partial D1-D3 28S region using LSU5 (Littlewood, 1994), 300F (Littlewood et al., 2000), ECD2 (Littlewood et al., 1997) and 1500R (Snyder & Tkach, 2001) and the partial *cox*1 region using Dig_cox1Fa (Wee et al., 2017) and Dig_cox1R (Wee et al., 2017). Geneious® version 10.2.6 (Kearse et al., 2012) was used to assemble and edit contiguous sequences, and the start and end of the ITS2 region were determined by annotation through the ITS2 Database using the ‘Metazoa’ model (Keller et al., 2009; Ankenbrand et al., 2015).

ITS2 and *cox*1 sequence data generated during this study were each aligned with MUSCLE in MEGA 7 (Kumar et al., 2016) using UPGMA clustering for iterations 1 and 2. Only two other schistorchiine ITS2 sequences were available on GenBank for inclusion in the analysis [AF392435–36; Lo et al. (2001)]; no comparable *cox*1 sequences were available. The ends of each ITS2 fragment were trimmed for a final dataset of 481 base positions (bp). The *cox*1 alignment was transferred to Mesquite v.3.31 (Maddison & Maddison, 2021), translated (echinoderm/flatworm mitochondrial code) and inspected for internal stop codons. After the correct reading frame was determined, the first column was then removed so that the reading frame began on position one, simplifying position-coding in downstream analyses. The final *cox*1 dataset was 474 bp. All codon positions in the *cox*1 dataset were evaluated for substitution saturation, as well as non-stationarity caused by base composition bias. Substitution saturation was assessed using the “Test of substitution saturation by Xia et al.” function (Xia et al., 2003; Xia & Lemey, 2009) as implemented in DAMBE v. 7.2 (Xia, 2018); no significant substitution saturation was detected. Non-stationarity was assessed using the χ2 function in PAUP v. 4.0 (Swofford, 2002); significant non-stationarity was not detected. Thus, all codons in the *cox*1 dataset were used in downstream analyses. Pairwise differences were estimated for each dataset using the following conditions: “Variance Estimation Method = None”, “Model/Method = No. of differences” and “Substitutions to Include = d: Transitions + Transversions” and “Gaps/Missing Data Treatment = Pairwise deletion”. Unrooted Neighbour joining analyses were conducted using MEGA 7 for each dataset to explore species boundaries, with the following parameters: “Model/Method = No. of differences”, “Substitutions to Include = d: Transitions + Transversions”, “Rates among Sites = Gamma Distributed” and “Gaps/Missing Data Treatment = Pairwise deletion”. Nodal support was estimated by performing 10,000 bootstrap replicates.

The partial 28S sequences generated during this study were aligned with sequences of related apocreadiids from GenBank (Table 1) using MUSCLE version 3.7 (Edgar, 2004) run on the CIPRES portal (Miller et al., 2010), with ClustalW sequence weighting and UPGMB clustering for iterations 1 and 2. The resultant alignment was refined using MESQUITE (Maddison & Maddison, 2021); the ends of the alignment were trimmed and ambiguously aligned regions removed, leaving a final trimmed dataset of 1,275 bp.

**Table 1:** Collection and accession data for Apocreadiidae species from GenBank incorporated in the 28S analyses

Species	Host species	GenBank accession #	Reference
**Apocreadiinae Skrjabin, 1942**			
*Callohelmis pichelinae* Cribb & Bray, 1999	*Hemigymnus melapterus* (Bloch)	FJ788495	Bray et al. (2009)
*Crassicutis cichlasomae* Manter, 1936	*Mayaheros urophthalmus* (Günther)	JQ389858	Pérez-Ponce de León et al. (2012)
*Crassicutis manteri* Pantoja, Scholz, Luque & Perez-Ponce de León, 2021	*Satanoperca jurupari* (Heckel)	MW692063	Pantoja et al. (2021)
*Homalometron armatum* (MacCallum, 1895) Manter, 1947	*Lepomis microlophus* (Günther)	KC710976	Curran et al. (2013a)
*Homalometron avis* Hernández-Mena, Cabañas-Granillo, Medina-Hernández & Pérez-Ponce de León, 2022	*Eugerres plumieri* (Cuvier)	ON814858	Hernandez-Mena et al. (2022)
*Homalometron cupuloris* (Ramsey, 1965) Cribb & Bray, 1999	*Lepomis microlophus*	KT823420	Fayton et al. (2016)
*Homalometron elongatum* Manter, 1947	*Gerres cinereus* (Walbaum)	HM038037	Parker et al. (2010)
*Homalometron frocioneae* Fayton & Andres in Fayton, Curran, Andres, Overstreet & McCallister, 2016	*Fundulus diaphanus* (Lesueur)	KT823419	Fayton et al. (2016)
*Homalometron manteri* (Overstreet, 1970) Cribb & Bray, 1999	*Leiostomus xanthurus* Lacepède	JX400852	Curran et al. (2013b)
*Homalometron octopapillatum* Perez-Ponce de Leon, Razo-Mendivil & Garcia-Magana, 2012	*Mayaheros beani* (Jordan)	JQ389863	Pérez-Ponce de León et al. (2012)
*Homalometron pallidum* Stafford, 1904	*Fundulus heteroclitus* (Linnaeus)	HM038043	Parker et al. (2010)
*Homalometron palmeri* Curran, Tkach & Overstreet, 2013a, 2013b	*Micropogonias undulatus* (Linnaeus)	JX400853	Curran et al. (2013b)
*Homalometron pseudopallidum* Martorelli, 1986	*Australoheros facetus* (Jenyns)	JX400856	Curran et al. (2013b)
*Homalometron robisoni* Fayton & McAllister in Fayton, Curran, Andres, Overstreet & McCallister, 2016	*Fundulus notatus* (Rafinesque)	KT823418	Fayton et al. (2016)
*Homalometron synagris* (Yamaguti, 1953) Cribb & Bray, 1999	*Scolopsis monogramma* (Cuvier)	AY222243	Olson et al. (2003)
*Neoapocreadium splendens* Cribb & Bray, 1999	*Scolopsis monogramma*	AY222242	Olson et al. (2003)
**Megaperinae Manter, 1934**			
*Haintestinum amplum* Pulis, Curran, Andres & Overstreet, 2014	*Acanthostracion quadricornis* (Linnaeus)	KF733447	Pulis et al. (2014)
*Megapera gyrina* (Linton, 1907) Manter, 1934	*Acanthostracion quadricornis*	KF733448	Pulis et al. (2014)
*Megapera orbicularis* (Manter, 1933) Manter, 1947	*Acanthostracion quadricornis*	KF733450	Pulis et al. (2014)
*Thysanopharynx elongatus* Manter, 1933	*Acanthostracion quadricornis*	KF733479	Pulis et al. (2014)
**Schistorchiinae Yamaguti, 1942**			
*Paraschistorchis zancli* (Hanson, 1953) Blend, Karar & Dronen, 2017	*Zanclus cornutus* (Linnaeus)	AY222240	Olson et al. (2003)
**Outgroup taxa**			
**Atractotrematidae Yamaguti, 1939**			
*Atractotrema sigani* Durio & Manter, 1969	*Siganus lineatus* (Valenciennes)	AY222267	Olson et al. (2003)
*Isorchis cannoni* Huston, Cutmore & Cribb, 2017	*Siganus lineatus*	MF803154	Huston et al. (2018)
*Isorchis currani* Andres, Pulis & Overstreet, 2016	*Selenotoca multifasciata* (Richardson)	MF803157	Huston et al. (2018)
**Haploporidae Nicoll, 1914**			
*Hapladena acanthuri* Siddiqi & Cable, 1960	*Acanthurus chirurgus* (Bloch)	MH244119	Andres et al. (2018)
*Hapladena nasonis* Yamaguti, 1970	*Naso unicornis* (Forsskål)	AY222265	Olson et al. (2003)

Bayesian inference and maximum likelihood analyses of the 28S dataset were conducted to explore relationships among these taxa. Bayesian inference analysis was performed using MrBayes version 3.2.7a (Ronquist et al., 2012) and maximum likelihood analysis using RAxML version 8.2.12 (Stamatakis, 2014), both run on the CIPRES portal. The best nucleotide substitution model was estimated using jModelTest version 2.1.10 (Darriba et al., 2012); both the Akaike Information Criterion (AIC) and Bayesian Information Criterion (BIC) predicted the TVM+I+Γ model as the best estimator and Bayesian inference and maximum likelihood analyses were conducted using the closest approximation to this model. Nodal support in the maximum likelihood analysis was estimated by performing 1000 bootstrap pseudoreplicates. Bayesian inference analysis was run over 10,000,000 generations (ngen = 10,000,000) with two runs each containing four simultaneous Markov Chain Monte Carlo (MCMC) chains (nchains = 4) and every 1,000th tree saved. Bayesian inference analysis used the following parameters: “nst = 6”, “rates = invgamma”, “ngammacat = 4”, and the priors parameters of the combined dataset were set to “ratepr = variable”. Samples of substitution model parameters, and tree and branch lengths were summarised using the parameters “sump burnin = 3,000” and “sumt burnin = 3,000”. Emprostiotrematids and haploporids were designated as outgroup taxa, based on relationships inferred in Pérez-Ponce de León & Hernandez-Mena (2019).

### Species recognition

Putative species were initially identified as operational taxonomic units (OTUs) based on both morphological and molecular distinctions. Morphological OTUs were assigned to genera using the classification of Blend et al. (2017). Molecular OTUs were distinguished based on nucleotide site similarity in each aligned *cox*1, ITS2 and 28S dataset. The final species recognition hypothesis is proposed relative to the criteria proposed by Bray et al. (2022), incorporating an integrated interpretation of morphology, host-specificity, and molecular data.

## Results

### Overview

Specimens consistent with the concept of the Schistorchiinae were collected from four families of the Tetraodontiformes (Balistidae, Monacanthidae, Tetraodontidae and Triacanthidae) and one of the Acanthuriformes (Zanclidae). Our analyses of further samples from scarids and siganids are inconclusive and those specimens, together with those of several rare species, are reserved for future publication dependent on further collections. Here we report on nine species, including two proposed as new.

### Morphology

Morphological examination distinguished nine relatively clear morphotypes, initially interpreted as relating to three of the genera recognised by Blend et al. (2017). Two known species of *Schistorchis* Lühe, 1906 are recognised: *Schistorchis carneus* Lühe, 1906 (the type-species) and *Schistorchis skrjabini* Parukhin, 1963. One known species of *Sphinteristomum* Oshmarin, Mamaev & Parukhin, 1961 is recognised: *Sphinteristomum acollum* Oshmarin, Mamaev & Parukhin, 1961 (the type-species). One species of *Lobatotrema* Manter, 1963 is recognised: *Lobatotrema aniferum* Manter, 1963 (the type- and only species); the genus and species are presently considered synonyms of *Sphinteristomum* and *Sph. acollum*. A new genus, *Blendiella* **n. gen.***,* is proposed for two new species consistent with the present diagnosis for *Schistorchis* Lühe, 1906 but for which we recognise subtle distinctions. The two new species are easily distinguished as novel, based on commonly invoked schistorchiine interspecific differences, especially testis number. Three known species of *Paraschistorchis* Blend, Karar & Dronen, 2017 are recognised: *Paraschistorchis stenosoma* (Hanson, 1953) Blend, Karar & Dronen, 2017; *P. seychellesiensis* (Toman, 1989) Blend, Karar & Dronen, 2017; and *P. zancli* (Hanson, 1953) Blend, Karar & Dronen, 2017. Seven of the species are reported from Australian waters for the first time.

### Molecular data

**Fig. 1:**
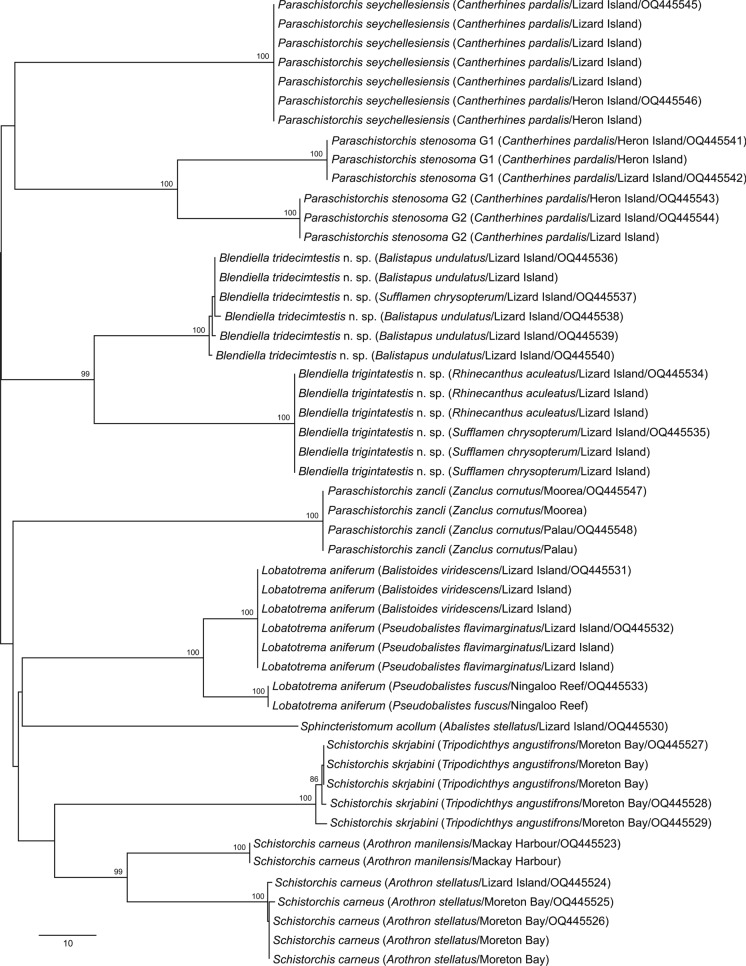

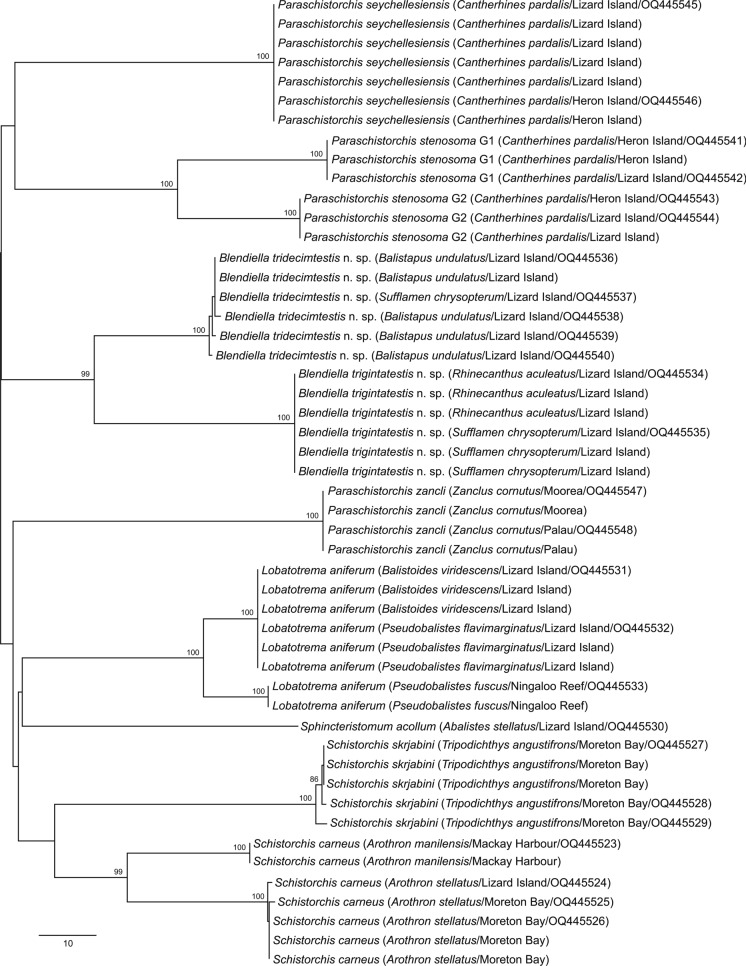
Phylogram from the unrooted Neighbour-joining analysis of the Schistorchiinae *cox*1 mtDNA dataset. Bootstrap support values are shown at the nodes, with values of <85 not shown. The scale-bar indicates the number of base differences. Abbreviations: G1, genotype 1; G2, genotype 2.

Neighbour joining analysis of the *cox*1 dataset distinguished the nine morphotypes listed above at 56–117 bp (Fig. 1). Replicate sequences were produced for eight of the species (for all except *Sph. acollum*) and, for five of these, intraspecific variation was insignificant, ranging from only 0–4 bp. In contrast, three morphotypes incorporated deep divisions in *cox*1 sequence data. Sequences from specimens consistent with *L. aniferum* from Ningaloo Reef and from off Lizard Island differed at 22 bp. Sequences from specimens consistent with *P. stenosoma* collected from *C. pardalis* from off both Heron and Lizard Islands differed at 48 bp. Sequences from specimens consistent with *Sch. carneus* from *Arothron stellatus* and *A. manilensis*, all from off the Queensland coast, differed at 48–49 bp.

**Fig. 2:**
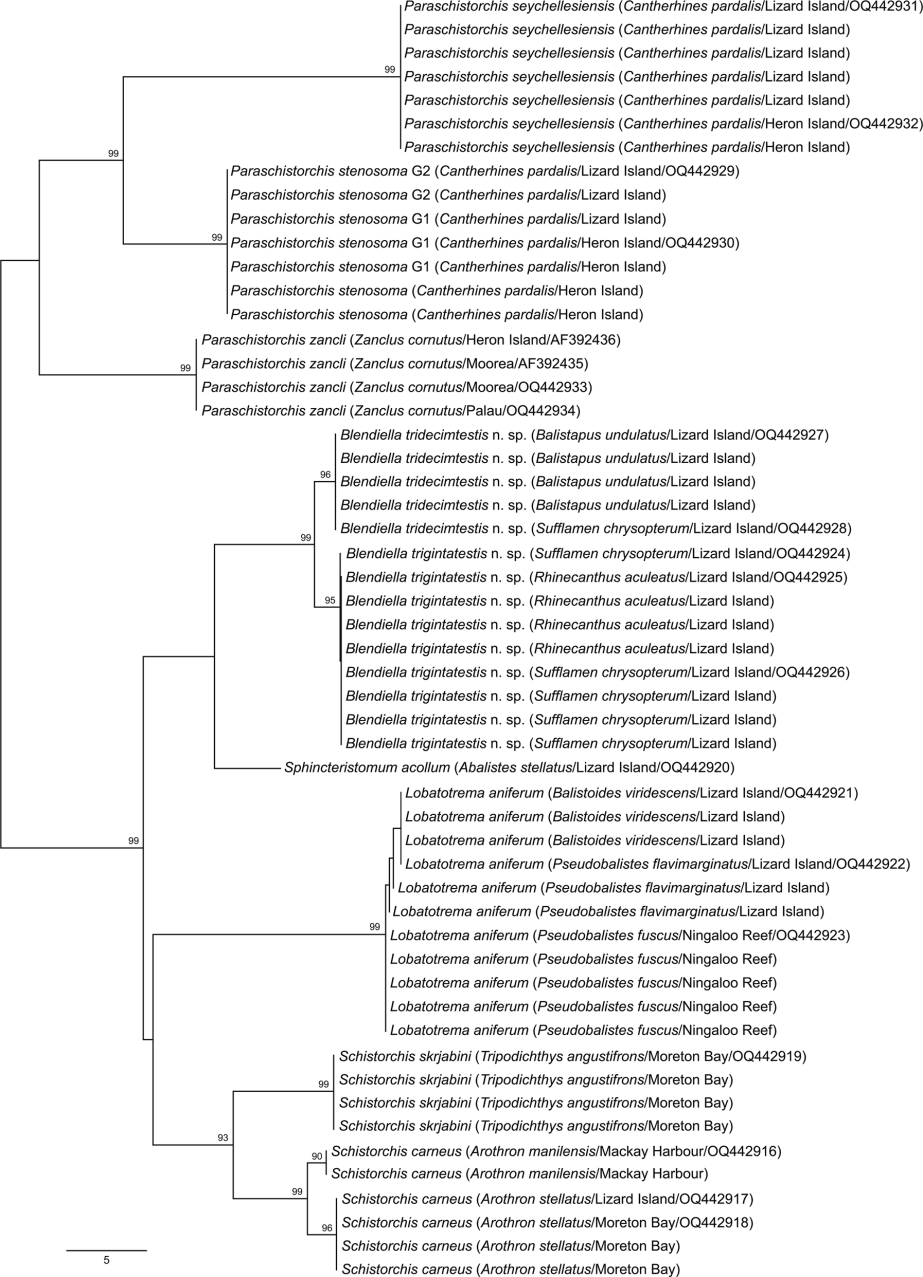
Phylogram from the unrooted Neighbour-joining analysis of the Schistorchiinae ITS2 rDNA dataset. Bootstrap support values are shown at the nodes, with values of <85 not shown. The scale-bar indicates the number of base differences. *cox*1 genotypes for *Paraschistorchis stenosoma* sequences shown where known. Abbreviations: G1, genotype 1; G2, genotype 2.

Neighbour joining analysis of the ITS2 dataset distinguished the nine morphotypes by 3–51 bp (Fig. 2). The two easily distinguishable species of *Blendiella* differed at only 3 bp in the ITS2 analysis whereas they differed at 56–57 bp in the *cox*1 analysis. The next most similar combination of putative species differed at 12 bp (*Sph. acollum* v. the two *Blendiella* species). The six ITS2 sequences relating to *P. stenosoma* were all identical, in contrast to the *cox*1 analysis in which they differed at 48 bp. The five sequences of *L. aniferum* from Ningaloo Reef differed from the six from off Lizard Island by just 1 bp in the ITS2 dataset, whereas they differed at 22 bp in the *cox*1 dataset. The four sequences relating to *Sch. carneus* from *A. stellatus* differed from the two from *A. manilensis* by 3 bp (the same as for the two species of *Blendiella*), whereas they differed by 48 bp in the *cox*1 analysis.

Twelve newly generated 28S rDNA sequences (selected based on distinctiveness in both *cox*1 and ITS2 datasets) were used principally to explore phylogenetic relationships (see below). In terms of species delineation, combinations of the nine morphospecies differed at 8–116 bp; the lowest distinction of 8 bp was between the two new species of *Blendiella*. The three morphospecies for which *cox*1 data demonstrated deep divisions (*L. aniferum, P. stenosoma*, and *Sch. carneus*) all had identical 28S sequences for corresponding specimens.

### Species recognition hypothesis

Delineation of the species considered here is relatively straightforward in the light of the species recognition criteria proposed by Bray et al. (2022). The concept of all nine morphotypes as representing distinct species is supported specifically by clear molecular distinctions and broadly by distinct host distributions. There are just three minor issues, all relating to *cox*1 variation between morphologically indistinguishable samples. In addition to the absence of meaningful morphological variation, for none of these combinations, with one possible exception, is there a meaningful host distinction. First, the variation in sequences from specimens consistent with *L. aniferum* from off Lizard Island and Ningaloo Reef is interpreted as intraspecific geographical variation of a kind that is now being reported frequently (e.g. McNamara et al., 2014; Cribb et al., 2022; Cutmore & Cribb, 2022; Wee et al., 2022). Secondly, the deep *cox*1 variation in sympatry seen for Great Barrier Reef (GBR) specimens consistent with *P. stenosoma* was not replicated in ITS2 or 28S rDNA sequences. We recognise both forms as a single species; comparable sympatric and morphologically indistinguishable populations have been reported for two species of *Preptetos* Pritchard, 1960 on the GBR (Bray et al., 2022). Thirdly, specimens morphologically consistent with *Sch. carneus* from off the Queensland coast create the greatest difficulty because distinctions in *cox*1 sequences were associated with specimens from different species of *Arothron*. Although this combination of evidence creates a *prima facie* case for recognition of two species, we conclude that it is based on too few specimens to justify the recognition of cryptic species and thus take the conservative approach of recognising a single species; the issue is considered in further detail below.

### Phylogenetic analysis

**Fig. 3:**
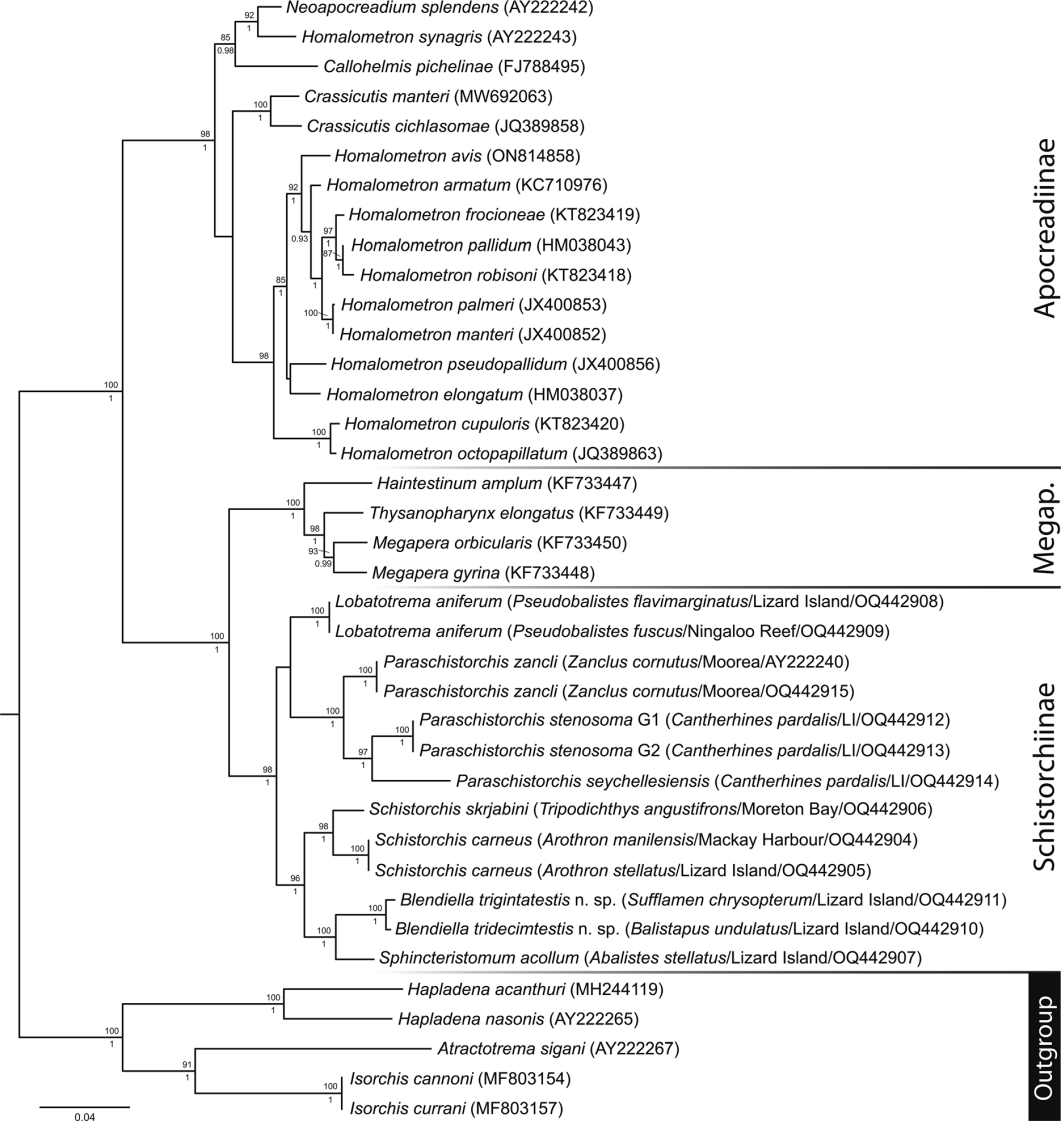
Relationships between species of the Apocreadiidae based on maximum likelihood phylogenetic analysis of the 28S dataset. Maximum likelihood bootstrap support values are shown above the nodes and Bayesian inference posterior probabilities shown below; values of <85 and <0.85 not shown. The scale-bar indicates expected number of substitutions per site. *cox*1 genotypes for *P. stenosoma* sequences shown. Abbreviations: G1, genotype 1; G2, genotype 2; LI, Lizard Island; Megap., Megaperinae.

Bayesian inference and maximum likelihood analyses of 28S rDNA dataset yielded identical topologies (Fig. 3), with robust support for the three apocreadiid subfamilies included in the analysis and the Schistorchiinae as sister to the Megaperinae. Within the schistorchiine clade, the type-species of the type-genus, *Sch. carneus*, formed a strongly-supported clade with *Sch. skrjabini*, but not with the two new species from Lizard Island balistids that have morphology broadly consistent with that of *Schistorchis* as diagnosed by Blend et al. (2017). Instead, these two species form a well-supported clade with *Sph. acollum* from which, however, they are dramatically morphologically distinct. A new genus, *Blendiella*, is proposed for them; morphological differences between the *Schistorchis* and *Blendiella* species are mainly subtle*.* Sequences of specimens here identified as *L. aniferum* from off Lizard Island and Ningaloo Reef formed a clade sister to all those mentioned above, quite distant from *Sph. acollum*, a species which it resembles and with which it has previously been synonymised. *Lobatotrema aniferum* is thus considered valid, rather than a synonym of *Sph. acollum*, and phylogenetic analysis supports the re-recognition of the genus *Lobatotrema.* Although these two species are morphologically distinguishable, the genus *Lobatotrema* is recognised as morphologically cryptic relative to *Sphincteristomum.* Finally, sequences relating to three species of *Paraschistorchis*, *P*. *seychellesiensis*, *P. stenosoma* and *P. zancli,* formed a strongly supported clade sister to all other sequenced schistorchiines.

In view of the revised genus-level classification mentioned above, we here propose a new key to the eight genera of the Schistorchiinae.

Key to genera of Schistorchiinae

1a. Testes two……………………………………………………………………………..…..2.

1b. Testes >two………………………..……………………………………………..………4.

2a. Caeca unite *via* uroproct……...…………...………….*Sphincterostoma* Yamaguti, 1937.

2b. Caeca open independently via separate ani…………………….…………………………3.

3a. Parasites of species of *Balistoides* and *Pseudobalistes* (Balistidae) ………...*Lobatotrema* Manter, 1963.

3b. Parasites of species of *Abalistes* and *Rhinecanthus* (Balistidae) and Sparidae…………………………....*Sphincteristomum* Oshmarin, Mamaev & Parukhin, 1961.

4a. Oral sucker highly glandular……………………………………………...……5.

4b. Oral sucker normally muscular, or not strongly glandular………………………………..6.

5a. Testes 8–11, normally in two distinct columns; body robust……. *Schistorchis* Lühe, 1906.

5b. Testes ≥11, normally mainly in single column; body slender…………..*Blendiella ***n. gen.**

6a. Caeca open *via* uroproct.……….….…*Neomegacreadium* Machida & Kuramochi, 1999.

6b. Caeca terminate blindly or at separate ani……………………………….……………….7.

7a. Caeca end blindly……………………....*Plesioschistorchis* Blend, Karar & Dronen, 2017.

7b. Caeca open at marginal or submarginal ani………..……………………………… ……………………………………………….*Paraschistorchis* Blend, Karar & Dronen, 2017.

**Apocreadiidae Skrjabin, 1942**

**Schistorchiinae Yamaguti, 1942**

***Schistorchis *** Lühe, 1906

***Type-species*:** ***Schistorchis carneus*** Lühe, 1906**, by original designation**

### Diagnosis

With characters of Schistorchiinae *sensu* Blend et al. (2017). Body elongate to elliptical. Tegument spinous. Eye-spot pigment dispersed in forebody. Pre-oral lobe present. Oral sucker highly glandular, round in outline; U-shaped partial sphincter at aperture prominent. Ventral sucker round in outline, smaller than oral sucker. Oesophagus absent. Intestinal bifurcation immediately posterior to pharynx, anterior to ventral sucker. Intestinal caeca open via separate ani at posterior end of body. Testes normally 11 (rarely fewer), entire, extending posteriorly from near posterior margin of ventral sucker in median cluster or in two distinct elongate columns. Ovary entire, dextral or almost median in anterior hindbody, contiguous with anterior testes. Vitelline follicles distributed from near posterior extremity to anywhere from anterior hindbody to posterior forebody, confluent in post-testicular region. Excretory vesicle I-shaped, terminates close to posterior margin of posterior testis. Excretory pore terminal. In intestine of monacanthid, tetraodontid and triacanthid fishes in the Indo-Pacific.

*Type-species*: *Schistorchis carneus* Lühe, 1906.

*Other species*: *Schistorchis paruchini* Kurochkin, 1974; *Schistorchis skrjabini* Parukhin, 1963; *Schistorchis tetraodontis* (Nagaty, 1956) Blend, Karar & Dronen, 2017

***Schistorchis carneus*** Lühe, 1906

Syn. *Pleorchis oligorchis* Johnston, 1913

*Type-host*: *Arothron stellatus* (Bloch & Schneider), Stellate puffer (Tetraodontiformes: Tetraodontidae).

*Type-locality*: South Modragam Paar, Sri Lanka.

*New material*

*Hosts*: *Arothron manilensis* (Marion de Procé), Narrowlined puffer; *A. stellatus* (Tetraodontiformes: Tetraodontidae).

*Localities*: off Lizard Island (14°40'S, 145°27'E), northern GBR; Mackay Harbour (21°06'S, 149°13'E), north Queensland; Moreton Bay (27°25'S, 153°14'E), south-east Queensland, Australia.

*Site in host*: Intestine.

*Prevalence*: off Lizard Island: 1 of 3 (33%) *A. stellatus*. Mackay Harbour: 1 of 1 *A. manilensis*. Moreton Bay: 3 of 5 (60%) *A. stellatus*.

Deposition of specimens: QM G240397–406.

*Representative DNA sequences*: Partial *cox*1 mtDNA, seven sequences (four submitted to GenBank, OQ445523–26); ITS2 rDNA, six sequences (three submitted to GenBank, OQ442916–18); partial 28S rDNA, three sequences (two submitted to GenBank, OQ442904–05).

*Measurements*: Table 2.

**Table 2:** Measurements of *Schistorchis carneus*. Percentages are given as a proportion of body length.

Locality	Lizard Island	Moreton Bay	Mackay Harbour
*n*	2	2	2
Host	*A. stellatus*	*A. stellatus*	*A. manilensis*
Body L	5551–5558	8150–9136	7255–7579
Body W	2915–3231	3194–3473	2618–2624
Body W %	52.4–58.2	38.0–39.2	34.5–36.2
Forebody	1306–1477	1622–1663	1266–1449
Forebody %	23.5–26.6	18.2–19.9	17.5–19.1
Pre-oral L	79–81	262–348	85–90
OS L	857–1007	1133–1172	903–921
OS W	1102–1131	1101–1284	878–893
OS L %	15.4–18.1	12.4–14.4	11.9–12.7
Prepharynx L	0	0	0
Pharynx L	137–172	284–360	262–268
Pharynx W	461–530	537–639	429–459
VS L	519–526	637–766	504–537
VS W	411–478	679–710	546–606
VS L / OS L	0.52–0.61	0.54–0.68	0.55–0.59
VS W / OS W	0.36–0.43	0.55–0.62	0.61–0.69
VS to ant. testis	1008–1100	499–626	1050–1072
Testes no.	11	11	11
Ant. testis L	384–403	372–403	309–318
Ant. testis W	380–401	258–282	308–316
Posttest. L	1830–2501	3401–5054	4301–4406
Posttest. L %	33.0–45.0	41.7–55.3	58.1–59.3
Ovary L	206–242	472–485	443–465
Ovary W	266–277	362–497	424–426
Vitelline field L	3147–4131	6086–6971	5843–6076
Previtelline L	2111–2180	1831–1981	1799–1821
Postvitelline L	310–317	168–184	281–289
Egg L	72–85	70–87	75–80
Egg W	38–44	38–42	41–42

### Remarks

This species (the type-species of the type-genus of the Schistorchiinae) was described by Lühe (1906) from *Arothron stellatus* off Sri Lanka. It was subsequently reported from *Arothron hispidus* (Linnaeus) by Johnston (1913) as *Pleorchis oligorchis* in the family Fasciolidae Railliet, 1895, from an unspecified locality off north Queensland without reference to *Sch. carneus*. Odhner (1928) recognised *P. oligorchis* as a synonym of *Sch. carneus*, a proposal accepted by all subsequent workers. The species has also been reported by Hafeezullah (1981) from *A. hispidus* from the Gulf of Manaar, India and by Madhavi et al. (1986) from *Lagocephalus lunaris* (Bloch & Schneider) (Tetraodontidae) from the Bay of Bengal, India.

New specimens reported from the type-host, from the northern GBR and Moreton Bay, and from *A. manilensis* from off Mackay are morphologically consistent with previous descriptions of this species and are immediately distinct from all other described species of *Schistorchis* in their massive bodies. We conclude that the sequences reported here for specimens from the type-host (but not from the type-locality) serve to establish the molecular identity of the type-species of the type-genus for the Schistorchiinae.

Sequence data are available for specimens here identified as *Sch. carneus* from *A. stellatus* from off Lizard Island and from Moreton Bay and from *A. manilensis* from Mackay Harbour, almost midway between the two other sites. *cox*1 sequence data for samples from the two fish species differ at 48 bp, ITS2 sequence data differ at 3 bp, and 28S sequence data are identical. The 48 bp *cox*1 distinction, although substantial, is less than that between the most similar combination of species recognised here (56–57 bp), the two new species of *Blendiella*, and identical to the 48 bp difference between what is below interpreted as two sympatric populations of *P. stenosoma*. The ITS2 distinction of 3 bp is identical to that between the two species of *Blendiella*, and greater than any distinction interpreted as intraspecific (e.g. the two *cox*1 populations of *P. stenosoma* are identical). The absence of distinction between 28S sequences contrasts with a difference of 8 bp between the two species of *Blendiella* which are otherwise by far the most similar combination of species recognised in this data set for that marker. These data present a host-associated distinction in the molecular characterisation of these forms, but we have been unable to detect a consistent morphological difference between specimens from *A. manilensis* and *A. stellatus.* These data are problematic. Strictly, based on the species recognition criteria that we employ, combined molecular and host distinction creates a basis for the recognition of separate species. However, we choose not to propose a new species because of three qualitative weaknesses in the case. First, the molecular distinction is generally lower than that typically seen between combinations of species in this family. Secondly, the host distinction is marginal in that other records interpreted as *S. carneus* incorporate further host richness (*Arothron hispidus* and *Lagocephalus inermis*) which either casts doubt on the importance of host distinction or might suggest that even more unrecognised richness is present in the genus. Finally, there is only a single collection of two specimens from a single *A. manilensis*, so that there is negligible replication of the basis of the key distinction in the data set. We conclude that the conservative option is to identify all these specimens as *S. carneus* pending the gathering of further evidence.

***Schistorchis skrjabini*** Parukhin, 1963

*Type-host*: *Triacanthus biaculeatus* (Bloch), Short-nosed tripodfish (Tetraodontiformes: Triacanthidae) [as *Triacanthus brevirostris* Temminck & Schlegel].

*Type-locality*: South China Sea.

*New material*

*Hosts*: *Tripodichthys angustifrons* (Hollard), Black-flag tripodfish (Tetraodontiformes: Triacanthidae).

*Locality*: Moreton Bay (27°25'S, 153°14'E), south-east Queensland, Australia.

*Site in host*: Intestine.

*Prevalence*: 13 of 57 (23%).

*Deposition of specimens*: QM G240407–22.

*Representative DNA sequences*: Partial *cox*1 mtDNA, five sequences (three submitted to GenBank, OQ445527–29); ITS2 rDNA, four sequences (one submitted to GenBank, OQ442919); partial 28S rDNA, four sequences (one submitted to GenBank, OQ442906).

*Measurements*: Table 3.

**Table 3:** Measurements of *Schistorchis skrjabini* and *Sphincteristomum acollum*. Percentages are given as a proportion of body length.

	*Schistorchis skrjabini*	*Sphincteristomum acollum*
Locality	Moreton Bay	Lizard Island
*n*	11	1
Host	*T. angustifrons*	*A. stellatus*
Body L	1315–3651 (1959)	2126
Body W	456–789 (624)	1182
Body W %	21.6–41.4 (33.2)	55.6
Forebody	307–627 (399)	554
Forebody %	17.2–24.4 (21.1)	26.1
Pre-oral L	29–78 (44)	57
OS L	211–369 (272)	385
OS W	217–368 (278)	418
OS L %	10.1–17.2 (14.5)	18.1
Prepharynx	-	59
Pharynx L	41–106 (68)	118
Pharynx W	69–172 (115)	200
VS L	101–194 (130)	168
VS W	94–179 (130)	171
VS L / OS L	0.42–0.53 (0.48)	0.44
VS W / OS W	0.40–0.49 (0.47)	0.41
VS to ant. testis	contiguous	107
Testes no.	10–11 (10.9)	2
Ant. testis L	58–215 (130)	162
Ant. testis W	65–186 (120)	426
Post. testis L	-	224
Post. testis W	-	328
Posttest. L	541–1899 (870)	555
Posttest. L %	38.5–52.0 (43.8)	26.1
Ovary L	79–202 (123)	134
Ovary W	93–215 (141)	178
Vitelline field L	744–2924 (1473)	1618
Previtelline L	299–586 (390)	458
Postvitelline L	8–40 (27.5)	50
Egg L	45–62 (55)	67
Egg W	25–36 (31)	43

### Description (Fig. 4A)

**Fig. 4:**
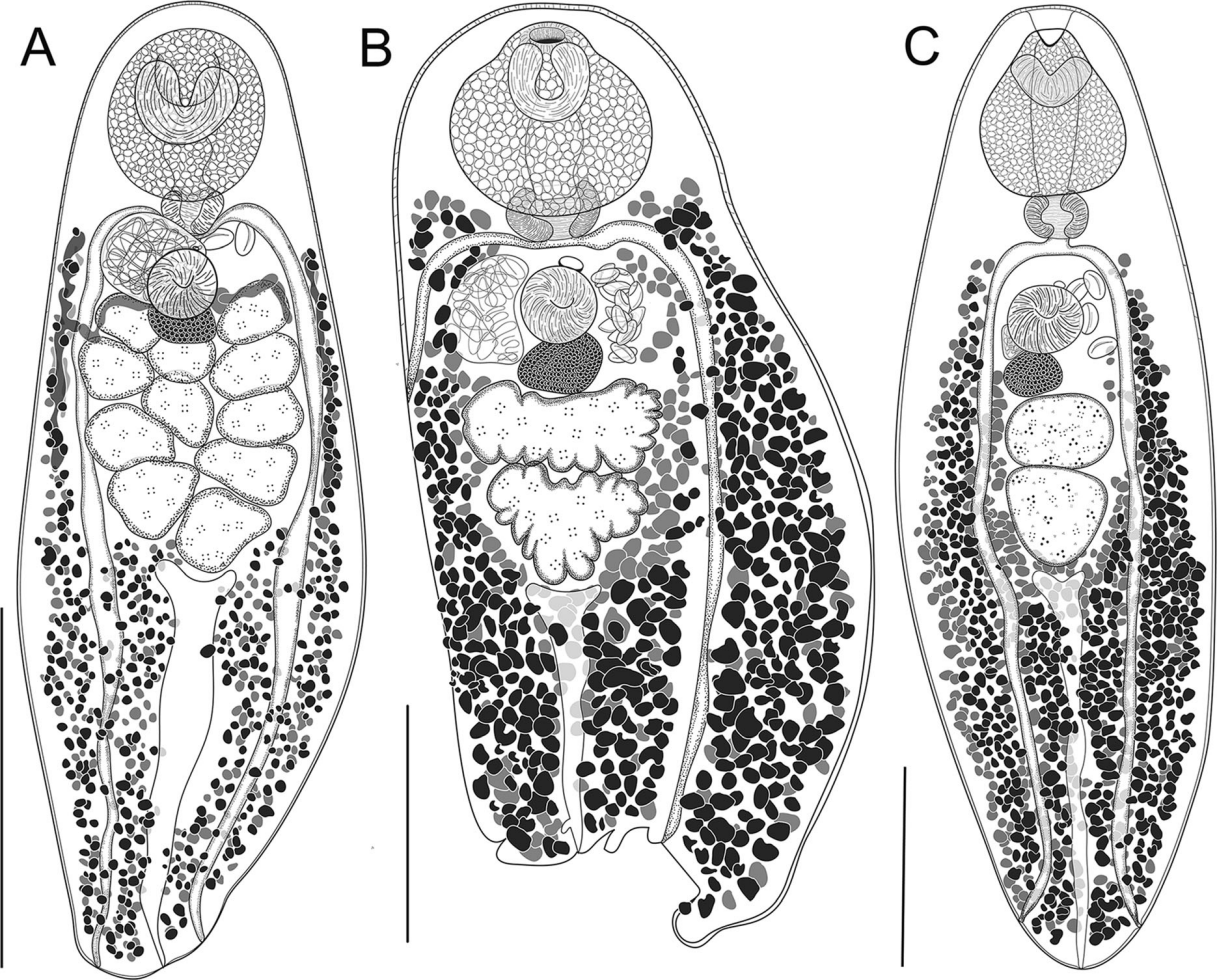
Species of Schistorchiinae from fishes of Queensland. A, *Schistorchis skrjabini* Parukhin, 1963 from *Tripodichthys angustifrons* from Moreton Bay, south-eastern Queensland; B, *Sphincteristomum acollum* Oshmarin, Mamaev & Parukhin, 1961 from *Abalistes stellatus* off Lizard Island, northern GBR; C, *Lobatotrema aniferum* Manter, 1963 from *Balistoides viridescens* off Lizard Island. *Scale-bars*, A, B, 500 µm; C, 250 µm.

[Based on 16 wholemount specimens, including 3 hologenophores.] Body robust, with maximum breadth in mid-hindbody and posterior end more sharply tapered than anterior end. Tegument spinous to level of ventral sucker. Pre-oral lobe strongly developed. Eye-spot pigment dispersed in forebody. Oral sucker round in outline, highly glandular; U-shaped muscular sphincter at oral aperture prominent. Prepharynx short. Pharynx unspecialised, rounded, typically partly dorsal to posterior margin of oral sucker. Oesophagus absent. Intestinal bifurcation immediately posterior to pharynx, close to level of anterior margin of ventral sucker. Caeca open at separate ani close to posterior extremity. Ventral sucker round in outline, unspecialised, far smaller than oral sucker. Testes 11 (10 in one specimen), entire, in compact group up to three testes wide and five testes long, close to ventral sucker; post-testicular region occupying approximately two-fifths to half body length. Seminal vesicle large, saccular, dextrally antero-dorsal to ventral sucker. Genital pore ventro-submedian, immediately anterior to ventral sucker. Ovary entire, ovoid, median, immediately anterior to most anterior median testis, usually partly overlaps ventral sucker dorsally. Canalicular seminal receptacle not detected. Vitelline follicles extend from level of ventral sucker or just entering forebody to close to posterior extremity, restricted to lateral margins anteriorly, occupying most available space posterior to testes, dorsally confluent immediately posterior to testes then separated into four variably distinct columns by intestinal caeca and excretory vesicle. Uterus short, passes from level of ovary to just anterior to ventral sucker. Excretory vesicle I-shaped, terminates at level of posterior margin of most posterior testis. Excretory arms extensive, to anterior margin of oral sucker. Excretory pore terminal.

### Remarks

This species was described by Parukhin (1963) from the South China Sea in the triacanthid *T. biaculeatus* (as *T. brevirostris*) and from the balistid *Abalistes stellatus* (Bloch & Schneider) [as *Abalistes stellaris* (Bloch & Schneider)]; *T. brevirostris* was the first-named host and is interpreted as the type-host here. The species was subsequently reported by Hafeezullah (1981) in *T. biaculeatus* (again as *T. brevirostris*) from the Bay of Bengal.

The specimens reported here are strongly consistent with the two previous descriptions of *Sch. skrjabini*. The specimens also resemble *Sch. paruchini* Kurochkin, 1974 from a monacanthid, *Meuschenia australis* (Donovan) [as *Navodon australis* (Donovan)], from the Great Australian Bight (Kurochkin, 1974). However, *Sch. paruchini* differs from *Sch. skrjabini* in having a relatively larger ovary and vitelline follicles and infecting a monacanthid (*vs* mainly triacanthids). The report of this species in a balistid is probably exceptional or erroneous. In Moreton Bay, *Sch. skrjabini* occurs commonly in *T. angustifrons* but in none of multiple monacanthids and tetraodontids examined there. However, we have examined no balistids from this location.

***Sphincteristomum*** Oshmarin, Mamaev & Parukhin, 1961

***Type-species: Sphincteristomum acollum*** Oshmarin, Mamaev & Parukhin, 1961**by original designation.**

### Diagnosis.

With characters of Schistorchiinae *sensu* Blend et al. (2017). Genus presently morphologically cryptic relative to *Lobatotrema* but phylogenetically distinct*.* Body ovate. Tegument spinous. Eye-spot pigment dispersed in forebody. Pre-oral lobe present. Oral sucker highly glandular, pyriform; U-shaped partial sphincter at aperture prominent. Ventral sucker round in outline, smaller than oral sucker. Oesophagus absent. Intestinal bifurcation immediately posterior to pharynx, anterior to ventral sucker. Intestinal caeca open via separate ani at posterior end of body. Testes two, distinctly lobed, in anterior hindbody. Ovary entire, dextral or almost median in anterior hindbody, contiguous with anterior testis. Vitelline follicles distributed from near posterior extremity to level of posterior margin of oral sucker, confluent in post-testicular region. Excretory vesicle I-shaped, terminates close to posterior margin of posterior testis. Excretory pore terminal. In intestine of balistid fishes (*Abalistes*) in the Indo-Pacific and sparid fishes in the Mediterranean Sea.

*Type-species*: *Sphincteristomum acollum* Oshmarin, Mamaev & Parukhin, 1961.

*Other species*: *Sphincteristomum mediterraneae* Abid-Kachour, Mouffok & Boutiba, 2013; *Sphincteristomum nikolevi* Parukhin, 1970.

***Sphincteristomum acollum*** Oshmarin, Mamaev & Parukhin, 1961

*Type-host*: *Abalistes stellatus* (Bloch & Schneider)*,* Starry triggerfish (Tetraodontiformes: Balistidae).

*Type-locality*: Tonkin Bay, Vietnam.

*New material*

*Host: A. stellatus.*

*Locality*: off Lizard Island (14°40'S, 145°27'E), northern GBR, Australia.

*Site in host*: Intestine.

*Prevalence*: 1 of 1 (100%).

*Deposition of specimens*: Hologenophore QM G240423.

*Representative DNA sequences*: Partial *cox*1 mtDNA, one sequence (submitted to GenBank, OQ445530); ITS2 rDNA, one sequence (submitted to GenBank, OQ442920); partial 28S rDNA, one sequence (submitted to GenBank, OQ442907).

*Measurements*: Table 3.

### Description (Fig. 4B)

[Based on a single whole-mounted hologenophore from *A. stellatus* from off Lizard Island.] Body broad, with maximum breadth in mid-hindbody; posterior margin damaged. Tegument spinous to mid-hindbody. Pre-oral lobe distinct. Eye-spot pigment dispersed in forebody. Oral sucker roughly pyriform, highly glandular; U-shaped muscular sphincter embedded at aperture. Prepharynx short. Pharynx large, rounded, with anterior portion dorsal to oral sucker. Oesophagus absent. Intestinal bifurcation just anterior to anterior margin of ventral sucker. One caecum opening at anus on damaged posterior margin; end of second caecum removed in portion used for sequencing. Ventral sucker round in outline, unspecialised, far smaller than oral sucker. Testes two, tandem, contiguous, in anterior hindbody, distinctly lobed; anterior testis significantly wider than long; posterior testis triangular; post-testicular region occupying approximately two-fifths body length (based on estimated full body length). Seminal vesicle saccular, prominent, dextral to ventral sucker. Genital pore ventro-submedian, slightly sinistral, immediately anterior to ventral sucker. Ovary ovoid, median, contiguous with anterior testis, mostly posterior to ventral sucker. Canalicular seminal receptacle not detected. Vitelline follicles extend from level of posterior margin of oral sucker to close to posterior extremity, mainly restricted to lateral margins anteriorly, occupying most available space around and posterior to testes, dorsally confluent immediately posterior to testes then broadly separated into columns by intestinal caeca and excretory vesicle. Uterus short, between level of ovary and genital pore. Excretory vesicle I-shaped, reaches to posterior testis. Excretory pore missing from damaged posterior extremity.

### Remarks

This species was described from *A. stellatus* (as *Abalistes stellaris*) from off Vietnam by Oshmarin et al. (1961) with two subsequent reports from the South China Sea (Parukhin & Chikunova, 1964; Parukhin, 1989), neither of which included a description or figure. Manter (1963) reported *Lobatotrema aniferum* from an unidentified balistid from Fiji with the species being synonymised with *Sph. acollum* by Yamaguti (1971). Machida & Kuramochi (1999) reported *Sph. acollum* from two other balistids, *Balistoides viridescens* (Bloch & Schneider) and *Pseudobalistes fuscus* (Bloch & Schneider), from off Japan. As discussed further following the report of the species, we consider *L. aniferum* valid and that the Japanese specimens should be identified as *L. aniferum* rather than *Sph. acollum*. In our view, *Sph. acollum* has only been reported from *A. stellatus.*

The single hologenophore reported here was incomplete at the time of collection, missing part of its posterior end, possibly from attack by other helminths. Apart from this damage, it is strongly consistent with the original species description. This report constitutes a new host and locality record for this species and the first record of it from Australia.

***Lobatotrema*** Manter, 1963

***Type-species*: *Lobatotrema aniferum*** Manter, 1963** by original designation.**

### Diagnosis.

With characters of Schistorchiinae *sensu* Blend et al. (2017). Genus presently morphologically cryptic relative to *Sphincteristomum* but phylogenetically distinct*.* Body elongate to elliptical. Tegument spinous. Eye-spot pigment dispersed in forebody. Pre-oral lobe present. Oral sucker highly glandular, pyriform; U-shaped partial sphincter at aperture prominent. Ventral sucker round in outline, smaller than oral sucker. Oesophagus absent. Intestinal bifurcation immediately posterior to pharynx, anterior to ventral sucker. Intestinal caeca open via separate ani at posterior end of body. Testes two, entire to distinctly lobed, in anterior hindbody. Ovary entire, dextral in anterior hindbody, contiguous with anteriormost testes. Vitelline follicles distributed from near posterior extremity to posterior forebody, confluent in post-testicular region. Excretory vesicle I-shaped, terminates close to posterior margin of posterior testis. Excretory pore terminal. In intestine of balistid fishes (*Balistoides*, *Pseudobalistes*) in the Indo-Pacific.

*Type- and only species*: *Lobatotrema aniferum* Manter, 1963.

***Lobatotrema aniferum*** Manter, 1963

Syn: *Sphincteristomum acollum* of Yamaguti (1971) in part and of Machida and Kuramochi (1999).

*Type-host*: “Balistid sp.” (Tetraodontiformes: Balistidae)

*Type-locality*: Off Fiji.

*New material*

*Hosts*: *Balistoides viridescens* (Bloch & Schneider), Titan triggerfish; *Pseudobalistes flavimarginatus* (Rüppell), Yellowmargin triggerfish; *Pseudobalistes fuscus* (Bloch & Schneider), Yellow-spotted triggerfish (Tetraodontiformes: Balistidae).

*Localities*: off Lizard Island (14°40'S, 145°27'E), northern GBR; Ningaloo Reef (21°55'S, 113°58'E), Western Australia, Australia. Off Îlot Goéland (22°22'S, 166°22'E), New Caledonia.

*Site in host*: Intestine.

*Prevalence*: off Lizard Island: 1 of 2 (50%) *B. viridescens*; 1 of 1 *P. flavimarginatus*. Ningaloo Reef: 4 of 7 (57%) *P. fuscus.* New Caledonia 1 of 1 *P. fuscus*.

*Deposition of specimens*: QM G240424–38; WAM V11697–712; MNHN HEL1900–4.

*Representative DNA sequences*: Partial *cox*1 mtDNA, eight sequences (three submitted to GenBank, OQ445531–33); ITS2 rDNA, 11 sequences (three submitted to GenBank, OQ442921–23); partial 28S rDNA, three sequences (two submitted to GenBank, OQ442908–09).

*Measurements*: Table 4.

**Table 4:** Measurements of *Lobatotrema aniferum*. Percentages are given as a proportion of body length.

Locality	Lizard Island	Lizard Island	New Caledonia	Ningaloo Reef
*n*	8	3	3	10
Host	*Balistoides viridescens*	*Pseudobalistes flavimarginatus*	*Pseudobalistes fuscus*	*Pseudobalistes fuscus*
Body L	1174–1412 (1250)	1674–1756 (1705)	1319–2151 (1802)	2120–3317 (2574)
Body W	393–489 (446)	497–566 (540)	462–713 (610)	755–1240 (971)
Body W %	33.1–39.1 (35.7)	28.3–33.8 (31.7)	33.1–35.6 (34.0)	30.8–42.2 (37.9)
Forebody	326–413 (358)	413–441 (423)	414–538 (462)	552–708 (622)
Forebody %	26.1–31.3 (28.6)	24.8–26.3 (24.8)	24.3–26.5 (25.2)	19.8– 29.2 (24.4)
Pre-oral L	19–47 (32)	35–42 (39)	38–60 (48)	61–97 (75)
OS L	195–222 (210)	212–262 (237)	245–296 (266)	268–398 (343)
OS W	161–183 (170)	183–223 (202)	174–255 (220)	242–397 (326)
OS L %	15.6–18.6 (16.8)	12.7–15.5 (13.9)	13.1–16.0 (14.3%)	10.9–16.5 (13.4)
Prepharynx L	19–30 (24)	25–29 (27)	-	-
Pharynx L	56–72 (63)	71–77 (74)	66–91 (78)	81–137 (109)
Pharynx W	88–99 (94)	96–116 (106)	108–149 (128)	144–211 (186)
VS L	82–112 (99)	96–125 (110)	98–142 (119)	140–212 (180)
VS W	96–121 (108)	106–147 (123)	107–166 (138)	151–251 (209)
VS L / OS L	0.41–0.53 (0.47)	0.45–0.48 (0.46)	0.43–0.48 (0.46)	0.47–0.56 (0.53)
VS W / OS W	0.58–0.71 (0.64)	0.53–0.66 (0.61)	0.61–0.65 (0.63)	0.61–0.71 (0.64)
VS to ant. testis	36–83 (65)	68–82 (76)	57–107 (82)	88164 (123)
Testis no.	2	2	2	2
Ant. testis L	79–132 (110)	114–144 (130)	106–154 (130)	185–317 (230)
Ant. testis W	128–162 (147)	203–231 (220)	173–277 (215)	338–522 (416)
Post. testis L	102–150 (119)	159–208 (175)	130–226 (173)	268–407 (316)
Post. testis W	106–152 (130)	177–215 (191)	164–251 (204)	305–469 (380)
Posttest. L	436–606 (517)	760–852 (792)	525–1064 (830)	855–1600 (1108)
Posttest. L %	37.0–43.9 (41.3)	45.1–48.5 (46.4)	39.8–49.5 (45.3)	35.9–49.0 (42.7)
Ovary L	50–70 (61)	70–94 (81)	83–126 (99)	112–142 (128)
Ovary W	69–84 (78)	109–141 (122)	95–164 (124)	164–227 (198)
Vitelline field L	810–1054 (914)	1284–1316 (1305)	944–1666 (1367)	1660–2768 (2051)
Previtelline L	270–377 (331)	378–422 (397)	387–477 (425)	438–683 (545)
Postvitelline L	9–17 (13)	21–50 (32)	26–84 (42)	14–91 (48)
Egg L	60	66–66 (66)	67–71 (69)	67–79 (72)
Egg W	31	32–32 (32)	32–40 (36)	32–40 (36)

### Description (Fig. 4C)

[Based on eight wholemount specimens, including two hologenophores from *B. viridescens* from off Lizard Island.] Body elongate linguiform, with maximum breadth at level of testes; posterior end slightly tapered, bluntly rounded. Tegument spinous in forebody. Pre-oral lobe distinct. Eye-spot pigment dispersed in forebody. Oral sucker ovoid to elongate pyriform, highly glandular; U-shaped muscular sphincter embedded at aperture. Prepharynx short. Pharynx subquadrate, immediately posterior to or slightly overlapping oral sucker. Oesophagus insignificant to absent. Intestinal bifurcation close to level of anterior margin of ventral sucker. Caeca open separately at posterior extremity at marginal ani. Ventral sucker round in outline, unspecialised, far smaller than oral sucker. Testes two, tandem, in midbody, contiguous, usually entire, sometimes distinctly lobed; post-testicular region occupying approximately two-fifths body length. Seminal vesicle saccular, small, dextral, mainly dorsal to ventral sucker. Genital pore immediately anterior to ventral sucker, slightly sinistrally submedian. Ovary ovoid, slightly dextral, contiguous with anterior testis, usually immediately posterior to ventral sucker. Canalicular seminal receptacle saccular, dorsal to ovary. Vitelline follicles extend from just anterior to ventral sucker, usually paralleling intestinal caeca, to close to posterior extremity, mainly restricted to lateral margins anteriorly, becoming more extensive posteriorly and occupying most available space around and posterior to testes, dorsally confluent immediately posterior to testes then broadly separated into four poorly defined columns by intestinal caeca and excretory vesicle. Uterus extends anteriorly from level of posterior margin of ovary. Excretory vesicle I-shaped, reaches to posterior testis. Excretory pore terminal.

### Remarks

*Lobatotrema aniferum* was described by Manter (1963) from an unidentified balistid from Fiji. The species was synonymised with *Sph. acollum* by Yamaguti (1971) following a personal communication with Manter who evidently agreed with the synonymy. The two species are highly similar, and we were first alerted to their apparent distinction by finding that relevant sequence data are unambiguously consistent with the presence of two species and, potentially, in terms of phylogenetic relationships, two genera. Overall, the combined morphological, molecular and host distribution evidence shows decisively that the new specimens reported here are distinct from *Sph. acollum* and consistent with the original description of *L. aniferum* by Manter (1963). Machida & Kuramochi (1999) reported specimens of *Sph. acollum* from Japan from two of the host species reported here. Although there are no supporting molecular data available, morphology and host distribution lead us to conclude that the Japanese specimens are best identified as *L. aniferum.*

**Fig. 5:**
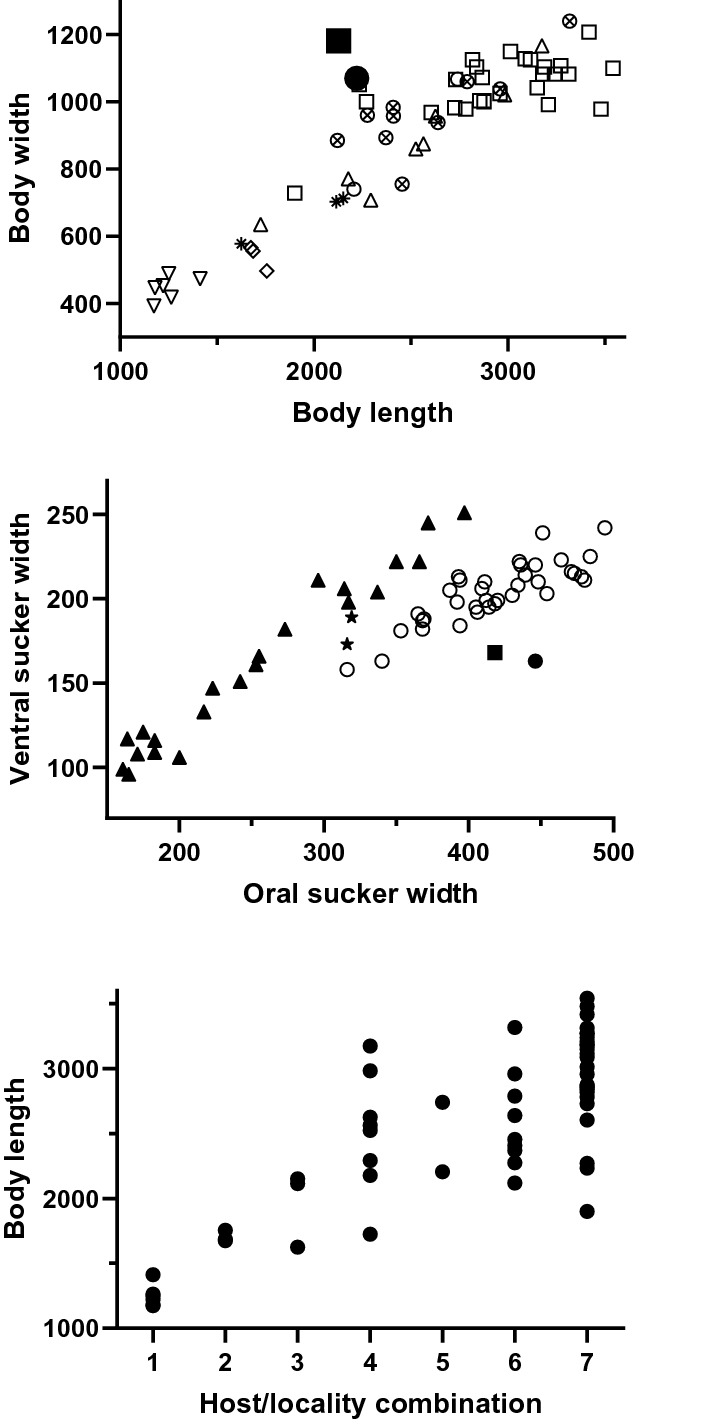
Morphometric comparisons of *Lobatotrema aniferum* Manter, 1963. A, Body length *vs* body width for *Sch. acollum* (● from original description by Oshmarin et al. (1961); ■ from *Abalistes stellatus¸* this study) and *L. aniferum* ( ◯ from description of *L. aniferum* from unknown balistid from Fiji by Manter (1963); □ from *B. viridescens* from Japan; △ from *P. fuscus* from Japan; ▽ from *B. viridescens* from Lizard island; ◊ from *P. flavimarginatus* from Lizard island; ⊗ from *P. fuscus* from Ningaloo Reef; ★ from *P. fuscus* from New Caledonia. B, Oral sucker width *vs* ventral sucker width for *Sch. acollum* ( ● from original description by Oshmarin et al. (1961); ■ from *A. stellatus¸* this study) and *L. aniferum* (★ from description of *L. aniferum* from unknown balistid from Fiji by Manter (1963); ◯ from *B. viridescens* and *P. fuscus* from Japan; ▲ all other specimens from Ningaloo Reef, the GBR and New Caledonia. C, Lengths of specimens of *L. aniferum* (1, *B. viridescens*, Lizard Island; 2, *P. flavimarginatus*, Lizard Island; 3, *P. fuscus*, New Caledonia; 4, *P. fuscus*, Japan; 5, unknown balistid, Fiji; 6, *P. fuscus*, Ningaloo Reef; 7, *B. viridescens*, Japan)*.*

The clearest indication of the distinction between *Sph. acollum* and *L. aniferum* is in the sequences generated from different balistid species in sympatry. *cox*1, ITS2 and 28S sequence data for these two species differ at 91–98, 18–19 and 65 bp respectively and, in phylogenetic analysis, the two species do not form a clade. In contrast, we found no reliable genus-level morphological distinctions. We considered the form of the oral sphincter, described originally as U-shaped for *Sph. acollum* and V-shaped for *L. aniferum*, but found no fundamental distinction in the specimens that we examined. The original description of *Sph. acollum* and our figured hologenophore specimen both have somewhat lobed testes whereas that in our figure of *L. aniferum* has smooth testes, but some of our specimens of *L. aniferum* have testes that are distinctly lobed, although perhaps not as strongly as in *Sph. acollum*. From morphometric analysis we found two distinctions between the two species. The body of specimens of *Sph. acollum* is relatively broader than that of *L. aniferum* (Fig. 5A); one of 38 Japanese specimens approaches the condition of *Sph. acollum* but otherwise the distinction seems reasonably clear. Charting of the widths of the oral and ventral suckers (Fig. 5B) suggests three groups of specimens – the two *Sph. acollum*, Japanese specimens, and those from Ningaloo Reef, the GBR, New Caledonia, and Fiji.

In summary, for specimens that we identify as representing *Sph. acollum* and *L. aniferum* we find unambiguous molecular distinction together with limited distinction based on morphology and host distribution. One morphological character suggests a possible distinction between Japanese specimens of *L. aniferum* and those from elsewhere, but we hesitate to identify them as distinct in the absence of any other evidence (molecular data or distinction in host). Notably, specimens from Japan were the largest examined (Fig. 5C), but specimens from Ningaloo Reef were almost as large. In the *cox*1 dataset, specimens from Ningaloo Reef and off Lizard Island form two distinct lineages, differing by 22 bp; ITS2 sequences for samples from the two localities differ at 1 bp and 28S sequences were identical. These molecular data are best interpreted as relating to geographical variation of a single species which may involve regional variation in overall size. In our view, the evidence suggests that *Lobatotrema* is best considered a “cryptic genus”; it is phylogenetically distinct but morphologically undifferentiated from the concept of *Schistorchis*. Whether it is useful to recognise such taxa is a complex issue; recognising cryptic genera may reflect biological reality whereas requiring morphological distinction maintains taxonomic utility. We find a small literature considering cryptic genera (Hsieh et al., 2014; Maggioi et al., 2018; Lehr et al., 2020), especially for Cyanobacteria (e.g. Shalygin et al., 2017; Pietrasiak et al., 2021) which might be predicted to be problematic given their limited morphological variability. Here we propose to recognise *Lobatotrema* because the available evidence suggests that it is real, an available name already exists for it, and to draw attention to what we suspect is a developing problem in trematode taxonomy as the molecular database expands for taxa with limited morphological variability (e.g. Yong et al., 2021). The generic diagnosis proposed above differs from that of *Sphincteristomum* only in the identified host range and in specific reference to phylogenetic distinction.

Relative to the two other species of *Sphincteristomum, L. aniferum* is easily distinguished from *Sph. nikolevi* Parukhin, 1970 (from a balistid, *Rhinecanthus* sp., from the Red Sea), which has opposite testes (Parukhin, 1970) and from *Sph. mediterraneae* [from a sparid, *Pagellus erythrinus* (Linnaeus) from the Mediterranean], which has the testes at the posterior end of the body (Abid-Kachour et al., 2013). *Sphincteristomum mediterraneae* is exceptional both in being reported from a sparid and in being the only schistorchiine species reported from outside of the Indo-west Pacific.

The evidence from New Caledonia, Ningaloo Reef, the GBR and Japan, suggests that *L. aniferum* is restricted to species of *Balistoides* Fraser-Brunner and *Pseudobalistes* Bleeker; unfortunately, the type-host of the species in Fiji was not identified beyond family. McCord & Westneat (2016) inferred phylogenetic relationships of the Balistidae, recognising three clades. Of the species of interest here, *P. fuscus* fell in Clade 1, *B. viridescens* and *P. flavimarginatus* fell as sister taxa in Clade 2, and the species of *Abalistes* (host of *Sph. acollum*) fell in Clade 3. These results indicated that neither *Balistoides* nor *Pseudobalistes* as presently constituted is monophyletic. The proposed solution included recognition of *P. fuscus* as a species of *Balistes* and *B. viridescens* as a species of *Pseudobalistes*. These changes do not appear to have been adopted in the fish literature, although we detect no literature presenting a dissenting view. Regardless, the balistid relationships reported by McCord & Westneat (2016) have the hosts of *L. aniferum* in two (non-sister) clades and those of *Sph. acollum* in the third. Unfortunately, these insights do not materially improve understanding of genus-level-distinction between *Lobatotrema* and *Sphincteristomum*.

***Blendiella*** **n. gen.**

### Diagnosis.

With characters of Schistorchiinae *sensu* Blend et al. (2017). Body relatively small, elongate with almost parallel sides. Tegument spinous. Eye-spot pigment dispersed in forebody. Pre-oral lobe present. Oral sucker highly glandular, pyriform; U-shaped partial sphincter at aperture prominent. Ventral sucker round in outline, smaller than oral sucker. Oesophagus absent. Intestinal bifurcation immediately posterior to pharynx, anterior to ventral sucker. Intestinal caeca open via separate ani at posterior end of body. Testes 10–33 (rarely <12), entire, in single column up to four testes wide anteriorly, usually narrowing to single testis wide posteriorly. Post-testicular region varying strongly in proportion with number of testes. Ovary entire, dextral in anterior hindbody, contiguous with anterior testis. Vitelline follicles distributed from near posterior extremity to anterior margin of ventral sucker or into forebody, confluent in post-testicular region. Excretory vesicle I-shaped, terminates anteriorly well posterior to posteriormost testis. Excretory pore terminal. In intestine of balistid fishes in the Pacific Ocean.

*Type-species*: *Blendiella trigintatestis* **n. sp.**

*Other species*: *Blendiella tridecimtestis* **n. sp.**

*Etymology*: The new name honours our colleague Professor Charles (Chuck) Blend and his extended contribution to this field, including specifically to the understanding of the Schistorchiinae.

*ZooBank LSID*: urn:lsid:zoobank.org:act:3F9CF5AA-DCB0-4F70-AD47-51E865EEF228.

### Remarks

In the recent classification proposed by Blend et al. (2017), the two new species described below would fall unambiguously in *Schistorchis* on the basis of their highly glandular oral suckers and large numbers of testes. However, the molecular phylogenetic analyses presented here show that the two new species form a clade distant from the type-species of *Schistorchis*, *Sch. carneus*, which forms a strongly supported clade with one other sequenced species consistent with the present concept for the genus, *Sch. skrjabini*. If schistorchiine genera are to be maintained as monophyletic, several solutions to this problem are possible. *Schistorchis* and *Sphincteristomum* could be synonymised, recognising *Schistorchis* as a genus in which the testis number ranges from 2–33. We find that idea unsatisfactory for these parasites, although we note that Miller & Cribb (2013) took that approach in recognising *Siphomutabilis* Miller & Cribb, 2013 for cryptogonimids with two or multiple testes. *Megacreadium* Nagaty, 1956, presently a synonym of *Schistorchis*, could be re-recognised, but we see no basis to consider its poorly known type species, *M. tetraodontis* Nagaty, 1956, distinct from *Schistorchis sensu stricto* or as consistent with the two new species. Instead, we propose a new genus on the basis that the species included are not monophyletic with *Sch. carneus* and because we recognise some narrow morphological differences as follows. The two new species have 25–33 (29) (*B. trigintatestis*) and 10–16 (13) (*B. tridecimtestis*) testes whereas species of *Schistorchis* almost always have fewer (cf. *Sch. carneus* – 11 in all reports; *Sch. paruchini* – 11 in all reports; *Sch. skrjabini* – 11 in all reports; *Sch. tetraodontis* – 8 in single reported specimen). Just two of 19 *B. tridecimtestis* had fewer than 12 testes. The two new species are also significantly smaller and narrower than the four *Schistorchis* species. The largest specimen of the new genus is 1,631 µm long in comparison with recorded maxima of 14,400 µm for *Sch. carneus*, 2,900 µm for *S. kurochkini*, 3,600 µm for *S. skrjabini* and 13,500 µm for *Sch. tetraodontis*. The oral suckers of the two new species have a distinctive shape, constricted anteriorly, whereas those of species of *Schistorchis* are evenly round in outline. Finally, the two new species both infect balistids whereas the four recognised species of *Schistorchis* are reported from monacanthids, tetraodontids and triacanthids. We acknowledge that these distinctions are relatively subtle, but in combination they allow recognition of the new genus.

***Blendiella trigintatestis*** **n. sp.**

*Type-host*: *Sufflamen chrysopterum* (Bloch & Schneider), Halfmoon triggerfish (Tetraodontiformes: Balistidae).

*Other hosts*: *Rhinecanthus aculeatus* (Linnaeus), White-banded triggerfish; *Balistapus undulatus* (Park), Orange-lined triggerfish (Tetraodontiformes: Balistidae).

*Type-locality*: off Lizard Island (14°40'S, 145°27'E), northern GBR, Australia.

*Site in host*: Intestine.

*Prevalence*: 37 of 78 (47%) *S. chrysopterum*; 7 of 39 (18%) *R. aculeatus*; 13 of 19 (68%) *B. undulatus*.

*Deposition of specimens*: QM G240439–72.

*Representative DNA sequences*: Partial *cox*1 mtDNA, six sequences (two submitted to GenBank, OQ445534–35); ITS2 rDNA, nine sequences (three submitted to GenBank, OQ442924–26); partial 28S rDNA, two sequences (one submitted to GenBank, OQ442911).

*Measurements*: Table 5.

**Table 5:** Measurements of two new species of *Blendiella*. Percentages are given as a proportion of body length.

	*B. tridecimtestis*	*B. trigintatestis*		
Locality	Lizard Island	Lizard Island	Lizard Island	Lizard Island
*n*	19	8	12	7
Host	*Balistapus undulatus*	*Balistapus undulatus*	*Sufflamen chrysopterum*	*Rhinecanthus aculeatus*
Body L	1003–1397 (1234)	816–1631 (1258)	1227–1566 (1374)	816–1273 (1057)
Body W	210–335 (267)	214–343 (283)	237–311 (277)	214–290 (257)
Body W %	17.1–26.5 (21.7)	19.9–26.2 (22.8)	18.0–23.6 (20.2)	21.4–28.3 (24.5)
Forebody	187–253 (219)	191–273 (237)	203–248 (222)	191–246 (214)
Forebody %	15.7–19.3 (17.8)	15.8–23.4 (19.3)	15.1–17.8 (16.2)	16.7–23.4 (20.5)
Pre-oral L	15–37 (23)	25–42 (32)	19–29 (25)	18–24 (21)
OS L	108–137 (118)	96–126 (111)	101–117 (111)	96–117 (106)
OS W	117–162 (136)	112–163 (134)	115–139 (128)	112–133 (121)
OS L %	8.2–11.2 (9.6)	7.4–11.8 (9.1)	7.3–8.9 (8.1)	8.7–12.3 (10.2)
Prepharynx L	12–22 (16)	10–18 (14)	11–25 (14)	12–16 (14)
Pharynx L	28–46 (38)	29–39 (35)	27–45 (34)	28–34 (32)
Pharynx W	54–80 (64)	50–72 (60)	51–66 (60)	52–67 (59)
VS L	60–83 (71)	69–89 (76)	66–97 (78)	64–77 (70)
VS W	60–83 (74)	69–95 (81)	71–105 (84)	69–85 (76)
VS L / OS L	0.52–0.68 (0.60)	0.63–0.75 (0.68)	0.57–0.94 (0.71)	0.61–0.72 (0.67)
VS W / OS W	0.45–0.64 (0.60)	0.55–0.66 (0.60)	0.55–0.82 (0.66)	0.58–0.70 (0.63)
VS to ant. testis	48–98 (66)	46–101 (70)	67–104 (87)	50–81 (71)
Testis no.	10–16 (13)	28–32 (29)	25–32 (29)	25–33 (29)
Ant. testis L	22–42 (33)	28–45 (37)	26–47 (38)	28–44 (37)
Ant. testis W	25–48 (37)	26–50 (42)	32–58 (43)	41–48 (45)
Posttest. L	414–651 (502)	120–518 (301)	234–412 (325)	120–300 (202)
Posttest. L %	34.4–48.9 (40.6)	11.1–31.8 (23.0)	16.8–30.5 (23.8)	11.1–28.3 (19.5)
Ovary L	51–91 (71)	49–78 (61)	61–93 (81)	47–73 (58)
Ovary W	47–82 (68)	54–83 (65)	64–95 (75)	54–82 (62)
Vitelline field L	821–1196 (1024)	623–1391 (1023)	982–1331 (1140)	623–1039 (832)
Previtelline L	171–232 (204)	187–283 (246)	202–254 (226)	187–266 (226)
Postvitelline L	10–23 (16)	10–26 (19)	10–20 (14)	12–20 (15)
Egg L	47–65 (55)	50–66 (60)	52–70 (61)	57
Egg W	29–44 (34)	28–38 (33)	26–43 (33)	36

*ZooBank LSID*: urn:lsid:zoobank.org:act:86F228BA-4F4F-4FEF-B082-3CD8A82540E8.

*Etymology*: the specific name is composed from the Latin for 30 (*triginta*), and testis, in reference to the exceptional number of testes in this species.

### Description (Fig. 6A)

**Fig. 6:**
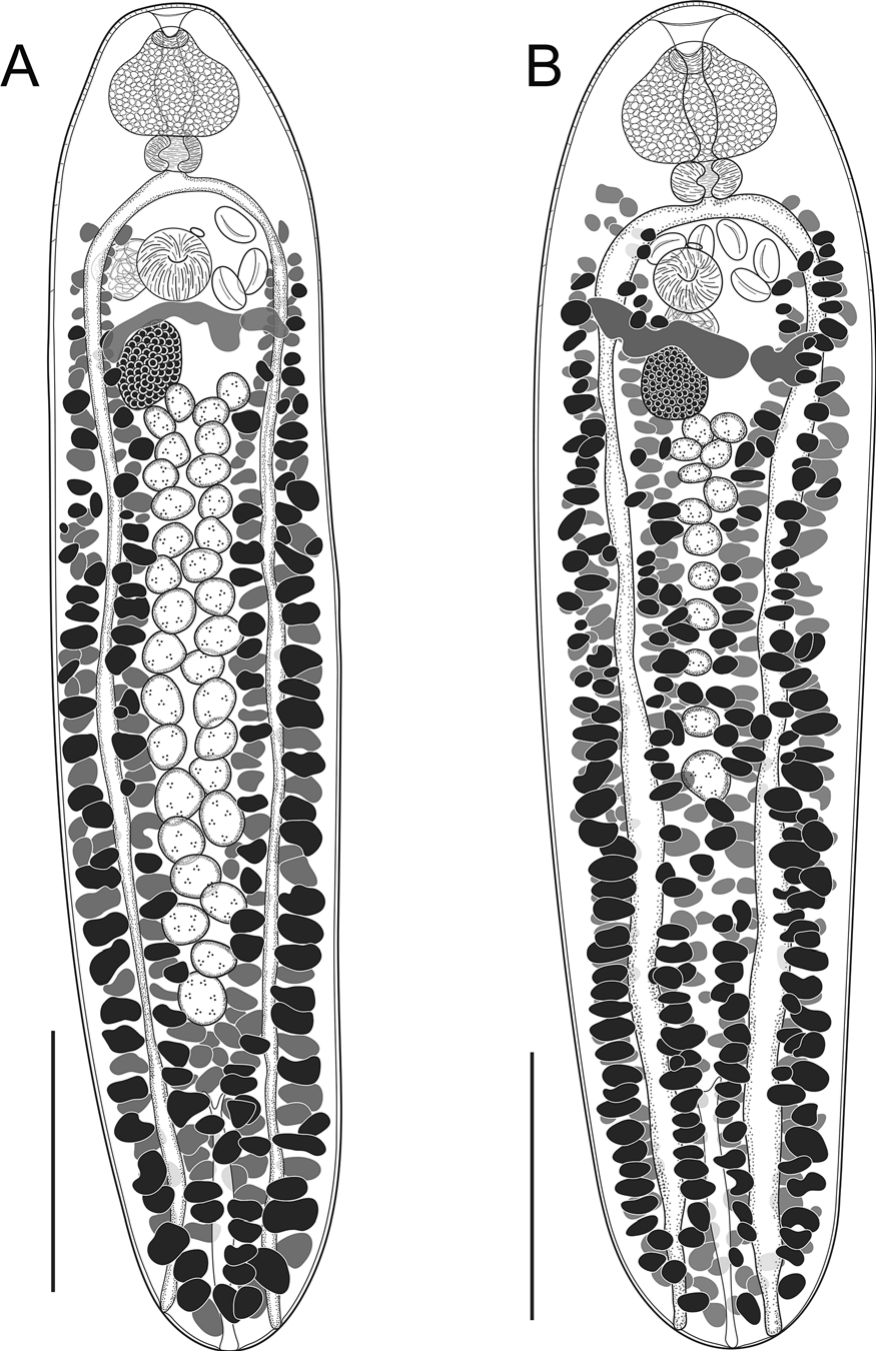
Species of *Blendiella* n. gen. from fishes off Lizard Island, northern Great Barrier Reef. A, *Blendiella trigintatestis* n. sp. from *Sufflamen chrysopterum*; B, *Blendiella tridecimtestis* n. sp. from *Sufflamen chrysopterum*. *Scale-bars*, A, B, 250 µm.

[Based on 14 wholemount specimens, including one hologenophore from *S. chrysopterum* from off Lizard Island.] Body small, elongate with nearly parallel margins, maximum breadth anywhere from level of ventral sucker to mid-hindbody; posterior end tapered and bluntly rounded. Tegument spinous in anterior forebody. Pre-oral lobe distinct. Eye-spot pigment dispersed in forebody. Oral sucker pyriform, with broad posterior margin, highly glandular; small U-shaped muscular sphincter embedded at aperture. Prepharynx short. Pharynx subquadrate, muscular and immediately posterior to oral sucker, sometimes dorsally overlapping oral sucker slightly. Oesophagus absent. Intestinal bifurcation in mid-forebody. Caeca open at separate marginal ani close to posterior extremity. Ventral sucker round in outline, unspecialised, far smaller than oral sucker. Testes 25–33, entire, contiguous, arranged in broad column from just posterior to ventral sucker to one-third of hindbody length from posterior extremity; column initially three or four testes wide, but narrows progressively, eventually to one posteriorly. Seminal vesicle small, saccular, dorso-dextral to ventral sucker. Genital pore immediately anterior to ventral sucker, slightly sinistro-submedian. Ovary roughly ovoid, posterior, and slightly dextral to ventral sucker, contiguous with anteriormost dextral testes. Canalicular seminal receptacle saccular, antero-dorsal to ovary. Vitelline follicles extend from just anterior to ventral sucker to close to posterior extremity, mainly paralleling intestinal caeca anteriorly, becoming more extensive posteriorly and occupying most available space around and posterior to testes, confluent immediately posterior to testes then broadly separated into four poorly defined columns by intestinal caeca and excretory vesicle. Uterus short, restricted to area between ovary and pharynx. Excretory vesicle I-shaped, relatively short, terminates well short of posterior testis. Excretory pore terminal.

### Remarks

This species is unique within the concept of the Schistorchiinae in the high number of testes (25–33). For both this and the following species, many specimens were damaged, perhaps by partial predation by other helminths. In these incomplete specimens the testis number may be dramatically reduced, but the identity of the specimen is still usually recognisable by the organisation of the remaining testes.

***Blendiella tridecimtestis*** **n. sp.**

*Type-host*: *Balistapus undulatus* (Park), Orange-lined triggerfish (Tetraodontiformes: Balistidae).

*Other host*: *Sufflamen chrysopterum* (Bloch & Schneider), Halfmoon triggerfish (Tetraodontiformes: Balistidae).

*Type-locality*: off Lizard Island (14°40'S, 145°27'E), northern GBR, Australia.

*Site in host*: Intestine.

*Prevalence*: 8 of 19 (42.1%) *B. undulatus*; 1 of 78 (1.2%) *S. chrysopterum*.

*Deposition of specimens*: QM G240473–97.

*Representative DNA sequences*: Partial *cox*1 mtDNA, six sequences (five submitted to GenBank, OQ445536–40); ITS2 rDNA, five sequences (two submitted to GenBank, OQ442927–28); partial 28S rDNA, two sequences (one submitted to GenBank, OQ442910).

*Measurements*: Table 5.

*ZooBank LSID*: urn:lsid:zoobank.org:act:A6DB51BA-F5FC-4372-9ECF-C0F65B45C805.

*Etymology*: the specific name is composed from the Latin for 13 (*tridecim*) and testis in reference to the most common number of testes in this species.

### Description (Fig. 6B)

[Based on 30 wholemount specimens, including five hologenophores from *B. undulatus* from off Lizard Island]. Body relatively small, elongate with nearly parallel margins, with maximum breadth anywhere from level of ventral sucker to mid-hindbody. Posterior end tapered and bluntly rounded. Tegument spinous in anterior forebody. Pre-oral lobe distinct. Eye-spot pigment dispersed in forebody. Oral sucker pyriform, with broad posterior margin, highly glandular; small U-shaped muscular sphincter embedded at aperture. Prepharynx short. Pharynx subquadrate, muscular and immediately posterior to oral sucker, sometimes dorsally overlapping oral sucker slightly. Oesophagus absent. Intestinal bifurcation in mid-forebody. Caeca open at separate marginal ani close to posterior extremity. Ventral sucker round in outline, unspecialised, far smaller than oral sucker. Testes 11–16, entire, contiguous anteriorly, slightly separated posteriorly, arranged in broad column from distinctly posterior to ventral sucker to mid-hindbody; column initially two or three testes wide but rapidly narrows progressively to one for posterior half. Seminal vesicle small, saccular, dorso-dextral or dorso-posterior to ventral sucker. Genital pore immediately anterior to ventral sucker, sightly sinistro-submedian. Ovary roughly ovoid, posterior to (sometimes contiguous with, sometimes slightly separated from) and slightly dextral to ventral sucker, contiguous with anteriormost testes. Canalicular seminal receptacle saccular, antero-dorsal to ovary. Vitelline follicles extend from about level of pharynx to close to posterior extremity, mainly paralleling intestinal caeca anteriorly, becoming more extensive posteriorly and occupying most available space around and posterior to testes, confluent immediately posterior to testes then broadly separated into four columns by intestinal caeca and excretory vesicle. Uterus short, restricted to area between ovary and pharynx. Excretory vesicle I-shaped, relatively short, terminates well short of posterior testis. Excretory pore terminal.

### Remarks

This species is easily distinguished from its congener by possessing only 11–16 (*vs* 25–33) testes. Relative to the four species of *Schistorchis* with which it shares multiple testes and a glandular oral sucker, this is a distinctively small and narrow species. In addition, the testis number is almost always slightly higher than the maximum of 11 seen in those species (overlapping rarely), and the testes are principally in a single column whereas in the four species of *Schistorchis* they are mainly distributed lateral to each other.

This species is clearly closely related to *B. trigintatestis* with which it co-occurs in *B. undulatus*. However*,* there is an apparent distinction in host-specificity in that this species has been found overwhelmingly in *B. undulatus* whereas *B. trigintatestis* also occurs frequently in two other balistid species.

***Paraschistorchis*** Blend, Karar & Dronen, 2017

**Type*-species*: *Paraschistorchis stenosoma* (**Hanson, 1953**)**Blend, Karar & Dronen, 2017** by original designation.**

### Diagnosis

With characters of Schistorchiinae *sensu* Blend et al. (2017). Body elongate to elliptical. Tegument spinous. Eye-spot pigment dispersed in forebody. Pre-oral lobe present or inconspicuous. Oral sucker mainly muscular but some species with distinct glandular elements, round in outline; U-shaped partial sphincter at aperture prominent or almost undetectable. Ventral sucker round in outline, smaller than oral sucker. Oesophagus short but distinct. Intestinal bifurcation immediately anterior to ventral sucker. Intestinal caeca open via separate ani at posterior end of body. Testes normally 11 (rarely fewer), entire, anteriorly in column one or two testes wide, posteriorly reduces to single testis. Ovary entire, dextral or almost median in anterior hindbody, contiguous with anterior testis. Vitelline follicles distributed from near posterior extremity to anywhere from level of posterior margin of ovary to posterior forebody. Excretory vesicle I-shaped, terminates close to posterior margin of posterior testis. Excretory pore terminal. In intestine principally of monacanthid, siganid and zanclid fishes in the Pacific and Indian Oceans.

*Type-species*: *Paraschistorchis stenosoma* (Hanson, 1953) Blend, Karar & Dronen, 2017.

*Other species*: *Paraschistorchis longivesiculurus* (Hafeezullah, 1981) Blend, Karar & Dronen, 2017; *Paraschistorchis seychellesiensis* (Toman, 1989) Blend, Karar & Dronen, 2017; *P. zancli* (Hanson, 1953) Blend, Karar & Dronen, 2017.

***Paraschistorchis stenosoma*** **(**Hanson, 1953**)** Blend, Karar & Dronen, 2017

Syn. *Schistorchis stenosoma* Hanson, 1953

*Type-host*: *Cantherhines sandwichiensis* (Quoy & Gaimard) Sandwich Isle file [reported as *Cantherhines pardalis* (Rüppell), Honeycomb filefish] (Tetraodontiformes: Monacanthidae).

*Type-locality*: Hawaii, United States of America (USA).

*New material*

*Hosts*: *Cantherhines pardalis*; *Cantheschenia grandisquamis* Hutchins*,* Large-scaled leatherjacket (Tetraodontiformes: Monacanthidae).

*Localities*: off Lizard Island (14°40'S, 145°27’E), northern GBR; off Heron Island (23°27'S, 151°55'E), southern GBR, Australia.

*Site in host*: Intestine.

*Prevalence*: off Lizard Island: 3 of 9 (33%) *C. pardalis.* Off Heron Island: 17 of 35 (49%) *Cantherhines pardalis*; 26 of 43 (60%) *Cantheschenia grandisquamis.*

*Deposition of specimens*: Hologenophores Mitochondrial **Genotype A** QM G240498–500; vouchers not differentiated by mitochondrial sequences QM G240501–27.

*Representative DNA sequences*: Partial *cox*1 mtDNA, six sequences (four submitted to GenBank, OQ445541–44); ITS2 rDNA, seven sequences (two submitted to GenBank, OQ442929–30); partial 28S rDNA, four sequences (two submitted to GenBank, OQ442912–13).

*Measurements*: Table 6.

**Table 6:** Measurements of *Paraschistorchis stenosoma*. Percentages are given as a proportion of body length.

Locality	Heron Island	Heron Island
*n*	18	13
Host	*Cantherhines pardalis*	*Cantheschenia grandisquamis*
Body L	1629–3448 (2432)	1337–2875 (2083)
Body W	267–565 (421)	312–548 (411)
Body W %	13.8–22 (18.2)	17.1–23.3 (20)
Forebody	306–536 (426)	262–522 (376)
Forebody %	14.6–22.7 (18.3)	16.3–22.6 (18.3)
Pre-oral L	19–45 (35)	22–55 (36)
OS L	157–245 (204)	138–212 (177)
OS W	158–251 (214)	146–221 (181)
OS L %	6.6–10.6 (8.8)	7.4–10.3 (8.7)
Prepharynx L	23–46 (33)	19–37 (28)
Pharynx L	62–123 (94)	58–104 (81)
Pharynx W	68–157 (124)	90–154 (113)
VS L	115–461 (176)	116–189 (149)
VS W	128–544 (189)	121–204 (161)
VS L / OS L	0.71–0.84 (0.78)	0.76–0.90 (0.84)
VS W / OS W	0.70–0.86 (0.78)	0.82–0.94 (0.89)
VS to ant. testis L	117–449 (297)	139–329 (227)
Testis no.	10–11 (11)	11–11 (11)
Ant. testis L	52–142 (79)	59–134 (92)
Ant. testis W	75–166 (104)	95–191 (134)
Posttest. field L	59–1139 (627)	311–829 (598)
Posttest. field L %	15.6–33.0 (27.5)	23.3–28.8 (28.7)
Ovary L	70–176 (125)	84–152 (113)
Ovary W	80–178 (131)	85–145 (120)
Vitelline field L	905–2361 (1590)	805–1944 (1403)
Previtelline L	485–1038 (772)	457–909 (669)
Postvitelline L	13–585–(109)	21–85 (55)
Egg L	52–69 (60)	55–68 (61)
Egg W	30–39 (34)	30–37 (33)

### Description (Fig. 7A)

**Fig. 7:**
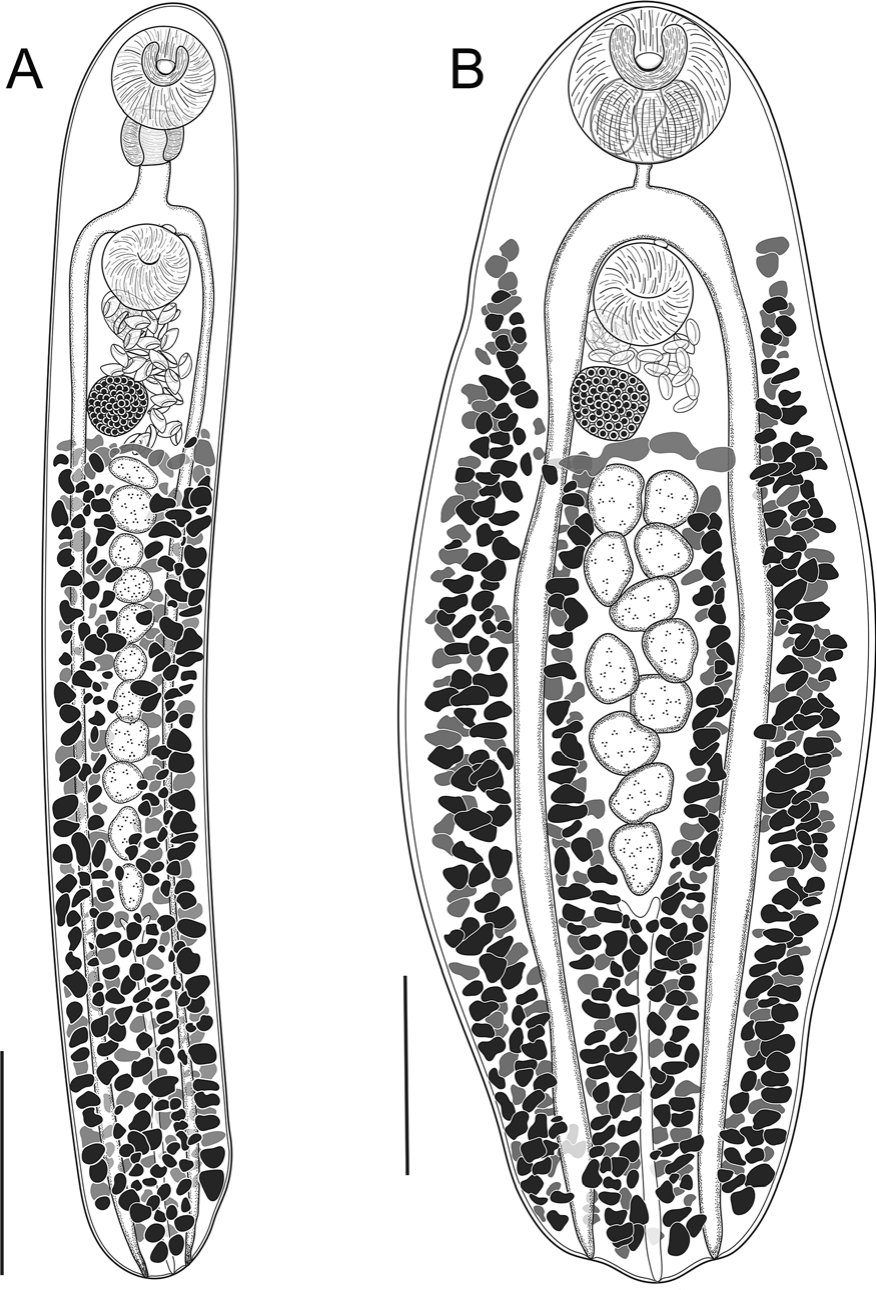
Species of *Paraschistorchis* from fishes of the Great Barrier Reef. A, *Paraschistorchis stenosoma* (Hanson, 1953) from *Cantherhines pardalis* off Heron Island; B, *Paraschistorchis seychellesiensis* (Toman, 1989) from *C. pardalis* off Heron Island. *Scale-bars*, A, B, 500 µm.

[Based on 18 wholemount specimens, including three hologenophores from *C. pardalis* from off Heron Island.] Body narrow, with almost parallel margins and maximum breadth usually slightly anterior to ventral sucker. Posterior margin rounded, roughly symmetrical relative to anterior margin. Tegument spinous to level of ventral sucker. Pre-oral lobe distinct. Eye-spot pigment dispersed in forebody. Oral sucker rounded in outline, muscular but with clear glandular components; U-shaped muscular sphincter embedded at aperture discernible, but not conspicuous. Prepharynx short. Pharynx subquadrate, typically partly dorsal to oral sucker. Oesophagus distinct. Intestinal bifurcation immediately anterior to ventral sucker. Caeca broad, terminate at separate ani at posterior extremity. Ventral sucker round in outline, unspecialised, only marginally smaller than oral sucker. Testes usually 11, (6, 7, 9 in each of one specimen), usually contiguous, commencing well posterior to ventral sucker, arrangement dependent on extension of body; in well-extended specimens testes in single column; in more contracted specimens, testes may be opposite anteriorly and zigzag posteriorly; post-testicular region occupying approximately one-quarter body length. Seminal vesicle saccular, small, postero-dorsal to ventral sucker. Genital pore ventro-submedian, immediately anterior to ventral sucker, slightly sinistral. Ovary rounded in outline, well-separated from and slightly dextral to ventral sucker. Canalicular seminal receptacle not detected. Vitelline follicles extend from about level of posterior margin of ovary to close to posterior extremity, occupying most available space around and posterior to testes, unevenly ventrally confluent posterior to testes, only weakly separated into columns by intestinal caeca and excretory vesicle. Uterus relatively long, extends slightly posterior to ovary, fills space between ovary and ventral sucker. Excretory vesicle I-shaped, reaches to posterior testis. Excretory pore terminal.

### Remarks

*Paraschistorchis stenosoma*, the type-species of *Paraschistorchis*, was originally described as *Sch. stenosoma* from *C. pardalis* off Hawaii by Hanson (1953). It was subsequently re-reported twice more from the same host/locality combination (Pritchard, 1963; Yamaguti, 1970). We infer that the fish species involved was actually *C. sandwichiensis*, a close relative of *C. pardalis* which occurs commonly at Hawaii, as *C. pardalis* does not occur in that region (Myers, 1991). Yamaguti (1970) also reported it from *Sufflamen fraenatum* (Latreille) (Balistidae).

*Paraschistorchis* presently comprises four species: *P. longivesiculurus*, *P. seychellesiensis*, *P. stenosoma* and *P. zancli*. Among these, *P. seychellesiensis* is easily distinguished as the broadest species. *Paraschistorchis longivesiculurus* is distinctive in having the testes reach close to the posterior extremity. *Paraschistorchis stenosoma* and *P. zancli* are the most similar species. Blend et al. (2017) invoked a distinction in the shape of the oral sucker (rounded to elliptical *vs* funnel-shaped), for these two species, but we have found the funnel-shape of *P. zancli* to be less distinctive in new specimens (reported below) than as reported originally by Hanson (1953). Instead, we find a clear difference in the sucker ratios and the anterior extent of the vitelline follicles between the two species. In addition to these morphological distinctions, the hosts (only or dominant) of *P. longivesiculurus* (Siganidae), *P. seychellesiensis* + *P. stenosoma* (Monacanthidae), and *P. zancli* (Zanclidae) appear to be reliably diagnostic. Our newly generated molecular data clearly distinguish *P. seychellesiensis*, *P. stenosoma* and *P. zancli*.

Considering the distinctions outlined above, the specimens reported from off Lizard and Heron Islands are reliably identifiable as *P. stenosoma*. Notably, in the *cox*1 dataset (Fig. 1), this species forms two sister lineages, differing at 48 bp, each occurring off both Heron and Lizard Islands; corresponding ITS2 and 28S sequences were identical (Figs. 2, 3). As discussed above, we interpret all these specimens as representing *P. stenosoma,* but we have identified the genotype of lodged voucher hologenophores to enable future consideration of the issue.

Our new records support the interpretation that *C. pardalis*, not *S. fraenatum*, is the main host for this species given that we have not detected it in 16 individuals of *S. fraenatum*, nor in five individuals of *S. bursa* (Bloch & Schneider) or 153 *S. chrysopterum*, examined from Australian waters. The present record is the first of this species from Australia.

***Paraschistorchis seychellesiensis*** **(**Toman, 1989**)** Blend, Karar & Dronen, 2017

Syn. *Schistorchis seychellesiensis* Toman, 1989

*Type-host*: *Cantherhines pardalis* (Rüppell), Honeycomb filefish [reported as *Cantherhines sandwichiensis* (Quoy & Gaimard, 1824) Sandwich Isle file] (Tetraodontiformes: Monacanthidae).

*Type-locality*: Seychelles Islands, Indian Ocean.

*New material*

*Host*: *C. pardalis*.

*Localities*: off Heron Island (23°27'S, 151°55'E), southern GBR; off Lizard Island (14°40'S, 145°27'E), northern GBR, Australia.

*Site in host*: Intestine.

*Prevalence*: off Lizard Island 8 of 9 (89%). Off Heron Island 14 of 35 (40%).

*Deposition of specimens*: QM G240528–41.

*Representative DNA sequences*: Partial *cox*1 mtDNA, seven sequences (two submitted to GenBank, OQ445545–46); ITS2 rDNA, seven sequences (two submitted to GenBank, OQ442931–32); partial 28S rDNA, two sequences (one submitted to GenBank, OQ442914).

*Measurements*: Measurements in Table 7.

**Table 7:** Measurements of *Paraschistorchis seychellesiensis* and *P. zancli*. Percentages are given as a proportion of body length.

	*P. seychellesiensis*	*P. zancli*
Locality	Heron Island, GBR	Moorea, FP
*n*	10	10
Host	*Cantherhines pardalis*	*Zanclus cornutus*
Body L	1120–4348 (2532)	1448–1973 (1681)
Body W	315–1301 (891)	244–306 (272)
Body W %	16.2–39.6 (30.8)	15.5–16.9 (16.2)
Forebody	246–891 (498)	333–454 (387)
Forebody %	16.2–25.1 (20.2)	18.9–28.6 (23.9)
Pre-oral L	18–63 (37)	19–41 (32)
OS L	137–413 (291)	113–185 (145)
OS W	146–406 (294)	137–179 (156)
OS L %	9.5–19.0 (12.1)	7.8–9.4 (8.6)
Prepharynx L	17–41 (32)	9–15 (12)
Pharynx L	50–191 (112)	70–90 (79)
Pharynx W	79–232 (156)	85–114 (109)
VS L	108–321 (205)	136–169 (155)
VS W	109–308 (207)	139–175 (159)
VS L / OS L	0.55–0.81 (0.71)	0.91–1.20 (1.11)
VS W / OS W	0.57–0.81 (0.71)	0.98–1.03 (1.01)
VS to ant. testis L	83–415 (247)	160–211 (182)
Testis no.	9–11 (10)	10–11 (11)
Ant. testis L	59–155 (111)	73–131 (101)
Ant. testis W	61–198 (114)	74–152 (121)
Posttest. L	235–1312 (785)	299–351 (322)
Posttest. L %	21.0–38.4 (30.5)	17.7–20.6 (19.2)
Ovary L	53–213 (139)	74–120 (95)
Ovary W	58–196 (137)	70–107 (89)
Vitelline field L	858–3238 (1802)	435–592 (504)
Previtelline L	390–962 (624)	544–611 (579)
Postvitelline L	35–182 (72)	39–48 (44)
Egg L	56–66 (62)	47–67 (55)
Egg W	34–39 (36)	33–37 (35)

### Description (Fig. 7B)

[Based on 13 wholemount specimens, including three hologenophores from *C. pardalis* from off Heron Island.] Body broad, linguiform, with maximum breadth at mid-hindbody; posterior margin sometimes indented slightly at each anus; anterior margin rounded. Tegument spinous to mid-forebody. Pre-oral lobe well developed. Eye-spot pigment dispersed in forebody. Oral sucker rounded in outline, muscular but with clear glandular components; U-shaped muscular sphincter embedded at aperture, well-developed and prominent. Prepharynx short. Pharynx small, subquadrate, typically partly dorsal to oral sucker. Oesophagus short but distinct in well-extended specimens. Intestinal bifurcation immediately anterior to ventral sucker. Caeca broad, terminate at separate ani at posterior extremity. Ventral sucker round in outline, unspecialised, noticeably smaller than oral sucker. Testes 9–11, roughly ovoid, in broad contiguous column, initially 2 testes wide but narrowing to one testis posteriorly; post-testicular region occupying approximately one-third body length. Seminal vesicle saccular, small, postero-dorsal to ventral sucker. Genital pore ventro-submedian, immediately anterior to ventral sucker, slightly sinistral. Ovary rounded in outline, dextral, typically slightly separated from ventral sucker anteriorly and anteriormost testis posteriorly. Canalicular seminal receptacle saccular, antero-dorsal to ovary. Vitelline follicles extend from about level of anterior margin of ventral sucker to close to posterior extremity, anteriorly principally lateral to intestinal caeca, posteriorly occupying most available space around and posterior to testes, unevenly ventrally confluent posterior to testes, separated into relatively distinct columns by intestinal caeca, testes, and excretory vesicle. Uterus short, restricted to area lateral and anterior to ovary to ventral sucker. Excretory vesicle I-shaped, reaches close to level of posterior testis. Excretory pore terminal.

### Remarks

This species was described by Toman (1989) from *C. pardalis* from off the Seychelles Islands in the Indian Ocean based on just one adult and one immature specimen. The present material is from the same host, broadly agrees with the original description of *P. seychellesiensis*, and is distinguished from the other three species by the breadth of the body. Notably, the original description shows the testes as forming a single column in contrast to the present specimens in which the column commences as two testes wide and narrows to one for the last few testes. This distinction translates into the post-testicular region in the original description occupying a considerably smaller proportion of total body length (16.3%) than in the present specimens, 21.0–38.4 (30.5)%. Whether this difference should be considered as intra-specific or inter-specific is debateable. Here we take the view that the similarities between these samples outweigh the differences and thus identify them as *P. seychellesiensis*. Clearly, further sampling and, ideally, sequencing from the type-location is necessary to improve confidence in this identification.

Despite the seemingly obvious differences between *P. stenosoma* and *P. seychellesiensis* as indicated by the two figures presented here, the two are not always as easily distinguished as might be expected. Inter-specific differences are most pronounced in the largest specimens. In these, the two species differ clearly in shape (*P. seychellesiensis* is much broader), oral sucker width (in specimens of equivalent length, that of *P. seychellesiensis* is significantly larger; the oral sucker of *P. stenosoma* never exceeds 251 µm in width in our samples but for *P. seychellesiensis* it ranges to 406 µm), the pharynx width tends to be larger in *P. seychellesiensis*, and the vitelline follicles commence further from the anterior end than do those of *P. seychellesiensis*. However, in specimens <2 mm long, all these distinctions converge. We recognised just one character distinction present in large easily distinguished specimens (including the hologenophores) that was also present in smaller specimens. The partial oral sucker sphincter of *P. seychellesiensis* is always discernible as a strongly and clearly recognisable structure whereas in *P. stenosoma* it is present, but far less easy to distinguish. Although the sphincters are here figured as comparable, that of *P. seychellesiensis* is consistently far better developed.

In molecular analysis, the present material consistently forms a single undivided clade for specimens from off Heron and Lizard Islands, in all *cox*1, ITS2 and 28S datasets (Fig. 1–3) and is clearly representative of a single species. This is the first record of this species from Australia.

***Paraschistorchis zancli*** **(**Hanson, 1953**)** Blend, Karar & Dronen, 2017

Syn. *Schistorchis zancli* Hanson, 1953

*Type-host*: *Zanclus cornutus* (Linnaeus), Moorish idol (Acanthuriformes, Zanclidae).

*Type-locality*: Hawaii, USA.

*New material*

*Host*: *Z. cornutus.*

*Localities*: off Heron Island (23°27'S, 151°55'E), southern GBR, Australia. Off Moorea (17°29'S, 149°51'W), Society Archipelago, French Polynesia. Off New Caledonia (22°34'S, 166°26'E). Off Palau (07°20'N, 134°29'E).

*Site in host*: Intestine.

*Prevalence*: off Heron Island 3 of 30 (10%). Off Moorea 9 of 15 (60%). Off New Caledonia 1 of 2 (50%). Off Palau 1 of 1 (100%).

*Deposition of specimens*: QM G240542–71.

*Representative DNA sequences*: Partial *cox*1 mtDNA, four sequences (two submitted to GenBank, OQ445547–48); ITS2 rDNA, two sequences (both submitted to GenBank, OQ442933–34); partial 28S rDNA, one sequence (submitted to GenBank, OQ442915).

*Measurements*: Table 7.

### Remarks

*Paraschistorchis zancli* was originally described from off Hawaii by Hanson (1953) from *Z. cornutus* based on two specimens, and was redescribed based on a larger sample collection from the same host/locality combination by Pritchard (1963). Yamaguti (1970) reported it from *C. sandwichiensis* (Monacanthidae) off Hawaii. Lo et al. (2001) reported and figured it from *Z. cornutus* from off Moorea (French Polynesia) and Heron Island (southern GBR) and reported identical 28S and ITS2 sequences and only inconsequential differences in morphology.

The new specimens are consistent with the previous morphological descriptions of the species with minor differences in appearance attributed to geographical or intraspecific variation. A novel observation here is that the supposedly key schistorchiine characteristic of a partial sphincter embedded in the oral sucker is exceptionally hard to detect. We note that the first two descriptions of the species (Hanson, 1953; Pritchard, 1963) made no mention of any such structure. Yamaguti (1970) noted that “its gaping aperture is provided with a ring of fine muscle fibers” but gave no figure. Lo et al. (2001) did not describe the oral sucker*.* Careful examination of multiple specimens from three localities (Heron Island, Palau, and French Polynesia) leads us to conclude that a sphincter is probably present but that, unlike in its congeners, it is exceptionally difficult to discern. We further note that, unlike in the congeners *P. stenosoma* and *P. seychellesiensis*, there is little if any development of gland cells in the oral sucker of this species.

Molecular data are identical for *cox*1 from French Polynesia and Palau and for ITS2 from French Polynesia, Heron Island and Palau (Figs 1–2); no fresh samples were available for sequencing from the GBR or New Caledonia.

## Discussion

### Genera of Schistorchiinae

As for most trematode taxa, generic diagnoses for the Schistorchiinae are presently based exclusively on adult morphological characters. To distinguish six genera of Schistorchiinae, Blend et al. (2017) required variation in only three characters (oral sucker glandular or muscular; number of testes; and the nature of the termination of intestinal caeca) to develop an effective system. However, because trematodes have a limited number of characters, morphology may fail to clearly reflect phylogeny reliably (Bray & Cribb, 2012) and thus lead to a classification that is not natural. Here we recognise two genera in addition to those recognised by Blend et al. (2017), both strongly supported as phylogenetically distinct but with relatively minor morphological distinction relative to the presently-recognised genera. Thus, it is concluded that the combination of two testes and intestinal caeca opening independently masks the phylogenetic distinction between species of *Sphincteristomum* and *Lobatotrema* and that the sharing of multiple testes and a highly glandular oral sucker obscures that between species of *Schistorchis* and *Blendiella.* In our view, the combination of morphological and molecular analyses results in a more nuanced and accurate classification, but not necessarily one that is easily applied

One of the key characters used by Blend et al. (2017) to distinguish schistorchiine genera, and still invoked by us, is the glandular *vs* muscular nature of the oral sucker. Our new scheme recognises the concepts of *Blendiella, Lobatotrema*, *Schistorchis* and *Sphincteristomum* as being partly defined by conspicuously glandular oral suckers and those of *Paraschistorchis*, *Plesioschistorchis*, *Neomegacreadium* Machida & Kuramochi, 1999 and *Sphincteristoma* as being partly defined by normally muscular oral suckers. We now suspect that this is an oversimplification. Here we have observed clearly glandular components in the oral suckers of two species of *Paraschistorchis* (*P. stenosoma* and *P. seychellesiensis*), although not nearly as pronounced as those of the species of *Blendiella, Lobatotrema*, *Schistorchis* and *Sphincteristomum*. In addition*,* we do not detect clear gland cells at all in the oral sucker of *P. zancli*. Strikingly, the sphincter in the oral sucker of *P. zancli* is reduced to the point that we have found it difficult to detect, although our view is that a weak structure is present. We hypothesise that glandular oral suckers and the oral sphincter are linked characters of the Schistorchiinae as a whole. We suggest a functionality whereby the sphincter allows host tissue to be securely captured in the cavity of the oral sucker where secretions of the gland cells begin some aspect of the digestion of the host tissue. If broadly correct, we would predict that the strongest gland cell development would be associated with strongly developed sphincters, and *vice versa* (as appears to be the case for *P. zancli*). This idea suggests that some level of glandular development may be expected in almost all schistorchiines although it need not obviate its careful use in the distinction of genera.

### Biogeography

Overall, what we know of the distribution of schistorchiine species fits a paradigm of broad Indo-west Pacific distributions of trematodes of tropical marine fishes (e.g. Lo et al., 2001; Chambers & Cribb, 2006; Cutmore et al., 2010; McNamara et al., 2012; Cutmore & Cribb, 2022). This conclusion is weakened by the fact that there is little distributional evidence based on molecular data. However, the data presently available (for *L. aniferum* and *P. zancli*) suggest that widespread distributions are plausible. In addition, the species under consideration are relatively morphologically distinctive. In this context there is evidence for the distribution of *Sch. carneus* in Sri Lanka, the Red Sea and eastern Australia, for *P. zancli* from Hawaii, French Polynesia, Palau and the GBR, for *L. aniferum* from Fiji, Japan, New Caledonia, the GBR and Ningaloo Reef, and for *Sph. acollum* from south-east Asia and the GBR. Presumably we can expect more, perhaps most species to prove to be widespread as collecting becomes more comprehensive.

Relative to the broad evidence for widespread species, there are several noteworthy exceptions. First, the two new species of *Blendiella* have both been found only from off Lizard Island on the northern GBR despite examination by us of a total of 145 individuals of their three host species elsewhere, including 87 from off Heron Island on the southern GBR. Evidently, not all the species occur everywhere that suitable hosts are found. Second, the two species known (mainly but not exclusively) from monacanthids of the genus *Cantherhines* show enigmatic distributions*. Paraschistorchis seychellesiensis* was described from the Seychelles and *P. stenosoma* was described and subsequently reported several times form Hawaii; neither species has been reported elsewhere. Here we found both species occurring in the same individual fish on the GBR, more-or-less halfway between the two original description sites. Molecular phylogenetic analysis finds that the two species are sister taxa. This relationship suggests the possibility that the two species diverged in allopatry (perhaps in cophyly with their definitive hosts, two species of *Cantherhines* although *P. stenosoma* also infects a species of *Cantheschenia*) and that their distributions subsequently expanded to now overlap. Such hypotheses are clearly speculative and require far more sampling, but the known patterns of distribution suggest that a range of processes may have affected the distributions of schistorchiines.

Finally, we draw attention to the varying patterns of intraspecific structuring suggested by *cox*1 sequences. This marker was sequenced for what we have interpreted as five species over ranges at least as great as that between Heron and Lizard Islands which are separated by about 1,200 km. Three patterns were recognised. *Paraschistorchis seychellesiensis* and *P. zancli* showed negligible *cox*1 distinctions over range. In contrast, *L. aniferum* differed at 22 bp between samples from Lizard Island and Ningaloo Reef, from opposite sides of the Australian continent. Finally, *S. carneus* and *P. stenosoma* were each represented on the GBR by two *cox*1 populations, both differing at 48 bp; in the case of *P. stenosoma*, both populations occur at both Heron and Lizard Islands. The differences of 48 bp are only marginally smaller than the 56–57 bp between the two new species of *Blendiella* which are easily differentiated by testis number but are otherwise highly similar. Contrasting patterns of regional population structure and its absence have been reported for related species of Aporocotylidae (Cutmore et al., 2021), Bivesiculidae (Cribb et al., 2022), Lepocreadiidae (Bray et al., 2018; 2022), and Monorchiidae (McNamara et al., 2014; Wee et al., 2022); we infer that such discrepancies should now come as no surprise. In contrast, the finding of distinct, sympatric *cox*1 lineages of the same species infecting the same fish species remains uncommon; it was reported for two species of the lepocreadiid genus *Preptetos* by Bray et al. (2022). In that study, and here, the two *cox*1 populations are interpreted as representing the same species on the basis of the species recognition criteria proposed by Bray et al. (2022); they fail the test for recognition as separate species because, although they form reciprocally monophyletic lineages, at a deeper level they are monophyletic, their hosts are the same, and they have indistinguishable morphology. Overall, we conclude that *cox*1 sequences frequently give insight into the recent population history of Indo-Pacific fish trematodes. The extent to which the inconsistency of *cox*1 similarity is a reliable reflection of population history remains to be explored.

## Data Availability

The data that support the findings of this study are available from the corresponding author upon reasonable request.
